# Trisomy 21 Disrupts Thyroid Hormones Signaling During Human iPSC-Derived Neural Differentiation In Vitro

**DOI:** 10.3390/cells14181407

**Published:** 2025-09-09

**Authors:** Janaina Sena de Souza, Sandra Sanchez-Sanchez, Nicolas Amelinez-Robles, B. S. Guerra, Gisele Giannocco, Alysson R. Muotri

**Affiliations:** 1 Department of Pediatrics, School of Medicine, University of California, San Diego, La Jolla, CA 90037, USA; sas018@health.ucsd.edu (S.S.-S.); bsiegelg@gmail.com (B.S.G.); amuotri@health.ucsd.edu (A.R.M.); 2 Department of Bioengineering, University of California, San Diego, La Jolla, CA 90037, USA; namelinezrobles@ucsd.edu; 3 Laboratory of Molecular and Translational Endocrinology, Division of Endocrinology, Department of Medicine, Escola Paulista de Medicina, Universidade Federal de São Paulo, São Paulo 04039-032, SP, Brazil; ggiannocco@unifesp.br; 4 Department of Biological Sciences, Universidade Federal de São Paulo-Campus Diadema, Diadema 09972-270, SP, Brazil; 5 Instituto de Ciências Ambientais, Químicas e Farmacêuticas, Universidade Federal de São Paulo, Diadema 09972-270, SP, Brazil

**Keywords:** down syndrome, thyroid hormones, transporters, deiodinase, receptors, gene expression, iPSC, astrocytes, neurons

## Abstract

Thyroid hormones (THs) are essential for brain development, and their dysregulation is associated with cognitive deficits and neurodevelopmental disorders. Down syndrome (DS), caused by trisomy 21, is frequently associated with thyroid dysfunction and impaired neurogenesis. Here, we investigated THs signaling dynamics during neural differentiation using human induced pluripotent stem cells (hiPSCs) derived from individuals with DS and controls. We analyzed the gene expression of key THs regulators—deiodinases, transporters, and receptors—and downstream target genes in hiPSCs, hiPSC-derived neural progenitor cells (NPCs), hiPSC-derived astrocytes, and hiPSC-derived neurons. DS-derived hiPSCs, hiPSC-derived NPCs, and hiPSC-derived neurons exhibited 2- to 7-fold increases in the gene expression of *DIO2* and 3- to 8-fold reductions in *DIO3*, alongside 1- to 3-fold downregulation of *THRA* and *THRB* isoforms. hiPSC-derived astrocytes showed a 4-fold decrease in the gene expression of *DIO2*, a 4-fold increase in *DIO3*, upregulation of *SLC16A10* (2-fold), and downregulation of *SLC7A5* (0.5-fold) and THs receptors (0.5- to 12-fold). hiPSC-derived neurons exhibited marked downregulation of the gene expression of *HOMER1* (0.5-fold), *GRIN3A* (14-fold), and *GRIN3B* (4-fold), accompanied by impaired spontaneous activity in multi-electrode array recordings. These findings reveal a robust, cell-type-specific imbalance between THs availability and signaling competence in DS hiPSC-derived neural cells, providing mechanistic insight into THs-related contributions to the function of DS hiPSC-derived neural cells and identifying potential therapeutic targets.

## 1. Introduction

Thyroid hormones (THs), primarily triiodothyronine (T3) and thyroxine (T4), are essential regulators that coordinate complex transformations across multiple tissues during post-embryonic development [1,2]. THs are also pivotal for maintaining metabolic balance throughout life, which is essential for brain development and allows organisms to adapt and thrive in diverse environmental conditions [3,4,5,6,7,8].

THs exert their effects by binding to THs receptors (THRs: TRα and TRβ), which regulate gene expression as ligand-dependent transcription factors [9]. Beyond this genomic pathway, THs influence gene expression through interactions with other transcription factors, signaling pathways, and epigenetic mechanisms, modulating histone acetylation and deacetylation via corepressors and coactivators and supporting the assembly of the transcription machinery. Additionally, THs elicit rapid non-genomic effects mediated by membrane receptors, such as the integrin αvβ3 [8,10,11].

THRs, particularly TRα1, are highly expressed in the brain and play a critical role in regulating genes involved in neurogenesis, myelination, and synaptic plasticity [9,12]. Recent studies suggest that T3 has a direct action on neurons via a novel neuronal pathway of T3 transport and action, increasing axonal T3 uptake into clathrin-dependent, endosomal/non-degradative lysosomes [13]. In adult humans, tissues with the highest T3 content include the brain, liver, and kidneys. Notably, the anterior pituitary and hypothalamus exhibit T3 levels that exceed those in plasma [14,15].

T3 action and signaling are critical for proper brain development and adult function, and disruptions in these processes can lead to significant cognitive deficits when they occur during critical developmental periods [16,17]. Dyshormonogenic primary congenital hypothyroidism, often caused by mutations in genes involved in thyroidal iodide transport, organification, or iodotyrosine synthesis and recycling, can impair THs production [18]. Additionally, disorders of THs signaling cause defects in membrane THs transporters, deficiencies in deiodinases (DIOs, enzymes responsible for THs metabolism), and resistance to THs due to pathogenic variants in the TRα or TRβ receptors [19,20]. Disruptions in THs synthesis, transport, metabolism, or signaling can have a severe impact on neurodevelopment, leading to irreversible neurological and cognitive impairments [18,21].

Thyroid dysfunction is commonly observed in individuals with Down syndrome (DS), a genetic condition resulting from trisomy of chromosome 21 [22]. DS presents diverse clinical phenotypes associated with cognitive impairment, developmental delays, an elevated risk of congenital heart defects, Alzheimer’s disease, and autoimmune thyroid disorders, particularly hypothyroidism [23,24]. Congenital hypothyroidism occurs in approximately 1% of individuals with DS, and abnormal thyroid function tests are reported in over 50% of neonates with DS [25,26,27].

For people with DS, the risk of developing thyroid disease persists throughout life, with autoimmune-related thyroid dysfunction becoming more prevalent with age [28]. In children with DS, subclinical hypothyroidism is the most frequent thyroid abnormality, often unrelated to autoimmune causes [29]. Most individuals with subclinical hypothyroidism who have DS receive L-T4 (levothyroxine) treatment. However, a small subset, particularly females with high levels of antithyroid autoantibodies, may develop overt hypothyroidism. This risk underscores the importance of regular thyroid function monitoring in individuals with DS to manage potential complications effectively [30].

At the molecular level, several genes located on chromosome 21 have been implicated in pathways that may disrupt TH signaling in DS. For example, overexpression of *Dyrk1a* is related to thyroid development dysfunction [31]. This gene, in humans, is triplicated in DS. Additionally, *SOD1* overexpression contributes to oxidative stress, which may cause THs homeostasis dysfunction [32]. These gene dosage imbalances may underlie the observed uncoupling between intracellular T3 availability and transcriptional responsiveness in DS hiPSC-derived neural cells, supporting a mechanistic link between trisomy 21 and TH signaling failure.

The high prevalence of thyroid dysfunction in DS suggests a possible connection between THs dysregulation and the disorder’s phenotypic features, warranting further research to explore this association.

Advances in stem cell technology have enabled the generation of hiPSCs from individuals with DS, allowing researchers to model DS-related pathologies in vitro, which provides a powerful tool to study disease mechanisms and develop targeted therapies [33]. These models offer an opportunity to explore the interplay between THs signaling and trisomy 21 at the cellular and molecular levels. For example, hiPSC-derived neurons from individuals with DS have revealed deficits in neurogenesis and synaptic function, which may be exacerbated by impaired THs signaling [34]. Furthermore, PSC-based systems can be used to test the efficacy of THs-based interventions in ameliorating deficits associated with DS.

In this study, we investigated how trisomy 21 alters THs signaling using hiPSC-derived neural cells. By analyzing the expression of key THs regulators and downstream targets across hiPSCs, hiPSC-derived neural progenitor cells, hiPSC-derived astrocytes, and hiPSC-derived neurons, we aimed to characterize cell-type-specific disruptions in THs pathways associated with DS.

## 2. Materials and Methods

### 2.1. Maintenance of Human iPSC

Human iPSCs were generated from dermal fibroblast cells isolated from a neurotypical control (1 cell line, laboratory repository) [35,36,37] and individuals with Down syndrome (2 cell lines, Coriell AG05397, AG06922) [35,36,38], as described before [37,38]. The hiPSCs were plated in 6-cm dishes coated with Matrigel (BD Biosciences, San Jose, CA, USA) and maintained in mTeSR plus medium (StemCell Technologies, Vancouver, BC, Canada). The medium was changed every other day, and the cells were split manually. This study was conducted in accordance with the guidelines of the Declaration of Helsinki, and the protocol was submitted to the Ethics Committee of the UCSD IRB/ESCRO (protocol 141223) on 28 February 2021, and subsequently reviewed and approved on 9 May 2024.

### 2.2. Generation of hiPSC-Derived Neuroprogenitor Cells, Neurons, and Astrocytes

We differentiated hiPSC-derived neural progenitor cells (hereinafter called hid-NPCs) from hiPSCs derived from DS samples (AG06922—DS1 and AG05397—DS2) and neurotypical controls. We maintained hiPSCs in mTeSR Plus medium (StemCell Technologies, Vancouver, BC, Canada) until achieving 80–90% confluency. Before differentiation, we treated the cells for one hour with fresh mTeSR Plus medium supplemented with 10 µM ROCK Inhibitor Y-27632 dihydrochloride (Ri) (Tocris). Subsequently, cells were dissociated into single-cell suspensions using a 1:1 mixture of Accutase and phosphate-buffered saline (PBS), then plated at a density of 4 × 10^6^ cells per well into a 6-well plate containing mTeSR Plus medium (StemCell Technologies) supplemented with 5 µM Y-27632, 10 µM Stemolecule SB-431542 (SB) (Stemgent, Cambridge, MA, USA), and 1 µM Dorsomorphin dihydrochloride (dorso) (Fisher Scientific). We maintained the cells in this medium for two days to induce embryonic body (EB) formation. After this, we changed the medium to STEMdiff Neural Induction Medium (NIM) (StemCell Technologies, Vancouver, BC, Canada) with SMAD inhibitors (SMADi, StemCell Technologies, Vancouver, BC, Canada). The culture plates were incubated at 37 °C with orbital shaking (95 RPM), with daily media replacement for five consecutive days. The EBs formed during this period were transferred onto Matrigel-coated 6 cm culture dishes and maintained for six additional days in NIM. Neural rosettes generated were mechanically dissociated into single-cell hid-NPC suspensions and subsequently plated onto poly-L-ornithine (10 µg/mL, Sigma-Aldrich) and laminin-coated (2.5 µg/mL, Gibco, Grand Island, NY, USA) 10 cm dishes. hid-NPC maintenance medium consisted of DMEM/F-12 (Thermo Scientific, Waltham, MA, USA) supplemented with 1X N2 NeuroPlex (Gemini), 1X Gem21 NeuroPlex (Gemini Bio-Products, Sacramento, CA, USA), and 1X penicillin/streptomycin (Thermo Scientific, Waltham, MA, USA) (NG medium), and 20 ng/mL of human FGF2 (Life Technologies, Carlsbad, CA, USA) was added to the NG medium every medium change.

To generate hiPSC-derived neurons (hereinafter called hid-Neurons), we expanded the hid-NPCs in NG medium supplemented with 20 ng/mL FGF2 until they reached approximately 75–85% confluency and then induced them to differentiate into hid-Neurons. Neuronal differentiation was initiated by treating cells with 10 µM Ri for 48 h without FGF2 supplementation. Subsequently, we changed the NG medium every 3 to 4 days. The hid-Neurons were considered mature after 6 weeks of differentiation.

To generate hiPSC-derived astrocytes (hereinafter called hid-Astrocytes), we induced sphere formation using fully confluent hid-NPC cultures, which we rinsed with PBS and then briefly incubated for 5 min at 37 °C in PBS. Following PBS removal, 9 mL of NG medium containing 20 ng/mL FGF2. The hid-NPC monolayer was detached mechanically using a cell lifter and dispersed via gentle pipetting. Cell suspensions (3 mL each) were transferred into individual wells of an ultra-low attachment 6-well plate and cultured under constant agitation (95 RPM) at 37 °C with 5% CO_2_.

On day two, we replaced the old medium with NG medium supplemented with 10 µM Ri for 48 h. Thereafter, we changed the medium to Astrocyte Basal Medium (ABM, Lonza, Basel, Switzerland), and cultures were maintained under continuous agitation for 15 days, with media renewal every 2 to 3 days.

At day 16, we plated the spheres onto culture dishes pre-coated with poly-L-ornithine (10 µg/mL, Sigma-Aldrich, St. Louis, MO, USA) and laminin (2.5 µg/mL, Gibco). Emerging hid-Astrocytes were enzymatically detached using Accutase, papain, and DNase and then expanded in ABM medium, with media changes performed every 2 to 3 days. The hid-Astrocytes were considered mature at passage 3.

### 2.3. Immunocytochemistry

The medium was aspirated, and we washed the cells with PBS. Then, we fixed them in 4% paraformaldehyde (PFA) for 15 min at room temperature (RT), followed by permeabilization with PBS containing 0.1% Tween-20 and blocking with PBS plus 2% BSA (Gemini) for 2 h at RT. Primary antibodies (iPSC: Nanog, OCT4, LIN28, SOX2; NPC: SOX2, MUS, SOX2, NES; Astrocyte: GFAP, AQP4, S100B; Neuron: MAP2, HOMER1, VGLUT1, NEUN, CTIP2) (Supplementary Table S1) were applied overnight at 4 °C and then incubated with secondary antibodies for 1 h at RT. Cells underwent three washes with PBS containing 0.1% Tween-20, and then we stained the nuclei with DAPI (Dako, Glostrup, Denmark). Coverslips were mounted onto slides using ProLong Gold mounting medium (Life Technologies, Carlsbad, CA, USA). We obtained the confocal images using a Zeiss fluorescence microscope equipped with Apotome (Axio Observer Apotome, Zeiss, Oberkochen, Germany).

### 2.4. Digital Karyotyping

We extracted genomic DNA from approximately 5 × 10^6^ cells using the QIAamp DNA Mini Kit (Qiagen, Germantown, MD, USA) according to the manufacturer’s instructions. We then evaluated the DNA quality and concentration using a NanoDrop spectrophotometer (Thermo Fisher Scientific, Waltham, MA, USA).

We sent the samples to the Institute for Genomic Medicine (UCSD) facility for digital karyotyping, which was performed to detect genomic copy number variations and structural alterations in cultured cells derived from hiPSCs.

### 2.5. RT-qPCR Assay

For RT-qPCR analysis, we extracted the total RNA using the RNeasy Mini Kit (Qiagen) according to the manufacturer’s instructions. We then assessed RNA quality and concentration with a NanoDrop spectrophotometer (Thermo Fisher Scientific). Approximately 1 µg of total RNA was reverse transcribed into cDNA using SuperScript III Reverse Transcriptase (Invitrogen, Carlsbad, CA, USA), following the manufacturer’s recommendations. We performed quantitative PCR using the SYBR Green PCR Kit (Qiagen, Germantown, MD, USA) on an ABI 7500 Fast Real-Time PCR System (Applied Biosystems, Foster, CA, USA). We designed primers for the characterization of hiPSCs, hid-NPCs, hid-Neurons, hid-Astrocytes, THs maintenance genes, Alzheimer’s disease-related genes, NMDA receptor subunits, and oxidative stress markers (Supplementary Table S2). We evaluated three different housekeeping genes and selected the most suitable reference gene for normalization based on the cell type analyzed. We quantified relative gene expression using the 2^−ΔΔCt^ method. We analyzed each sample in biological triplicate and technical duplicate.

### 2.6. Multi-Electrode Array

We obtained electrophysiological recordings using 48-well multi-electrode array (MEA) plates (Axion Biosystems, Atlanta, GA, USA), in each of which we plated 20,000 viable cells with 4-week-old hid-Neurons. We maintained cultures for 7 days in NG medium supplemented with 2% fetal bovine serum. After that, we kept the cells in NG medium, and cultured the neurons in BrainPhys medium two days before data acquisition.

We performed the recordings using the Maestro system (Axion Biosystems, Atlanta, GA, USA) and AxIS software (version 1.0, Axion Biosystems, Atlanta, GA, USA), with a 10 Hz–2.5 kHz filter and a dynamic threshold set at 5.5 times the noise standard deviation per electrode. Before recording, we equilibrated the plates for 2 min. We collected the data for 5 min and analyzed them using the Neural Metrics Tool (v2.5.1, Axion Biosystems, Atlanta, GA, USA). We considered active electrodes that registered 5 or more spikes per minute. Mean firing rates were derived from all active electrodes per subject. Network bursts were identified when ≥10 spikes appeared on ≥25% of active electrodes within a ≤100 ms inter-spike interval.

### 2.7. Proliferation Assay

To assess proliferation through direct cell counting, we plated 250,000 cells per well on poly-ornithine/laminin-coated 6-well plates. We evaluated each sample in three technical replicates. Following the specified culture duration, we dissociated the cells using Accutase for 5 min and resuspended them in an equal volume of DMEM/F12, and quantified using a Chemometec Via-1 cassette (Chemometec, Allerod, Denmark), which also determined the number of viable cells.

### 2.8. Cell Cycle Analysis

We enzymatically dissociated and counted hid-NPCs using the NucleoCounter NC-3000 with Via1-Cassettes (Chemometec, Allerod, Denmark). We then fixed the cells in 70% ethanol on ice or at 4 °C for a minimum of 2 h and incubated them at 37 °C for 5 min in a staining solution containing 0.5 mg/mL DAPI and 0.1% Triton X-100 in PBS. We loaded the samples onto NC-Slide A2 chambers (Chemometec, Allerod, Denmark), and fluorescence-based cell cycle profiling was performed following the manufacturer’s instructions.

### 2.9. Analysis of Publicly Available Single-Nucleus RNA Sequencing Data

#### 2.9.1. Gene Expression Dataset Acquisition

Differential gene expression values, including log2 fold change (Log2FC) values and adjusted *p*-values (padj), were obtained from the supplementary data of a published study in Proceedings of the National Academy of Sciences (PNAS) https://europepmc.org/article/MED/34795060 (accessed on 30 June 2025)). The dataset, provided as an Excel file, reports transcriptomic alterations across multiple cortical cell types from postmortem human brain samples analyzed via single-nucleus RNA sequencing. We utilized these processed data to infer expression patterns of genes relevant to THs signaling in hiPSC-derived neural cells. This analysis is strictly interpretative, based on statistically significant differential expression metrics as reported in the original publication. This analysis does not involve primary nuclei isolation or sequencing. All inferences are based on publicly available, pre-processed datasets curated by Palmer et al. (2021) [39].

#### 2.9.2. Data Preprocessing and Gene Categorization

Raw data corresponding to distinct cell types were merged into a unified R DataFrame. Genes of interest were curated and grouped into predefined biological categories: “Thyroid Hormones Markers,” “Alzheimer’s Disease-Related Genes,” “NMDAR Subunits,” and “Oxidative Stress-Related Genes.”

#### 2.9.3. Differential Expression Visualization

Volcano plots were generated to visualize gene-level differential expression. Log2FC values were plotted on the x-axis and –log10 (padj) on the y-axis. Thresholds for statistical significance (|Log2FC| > 0.25; padj < 0.05) were marked with dashed lines. Genes of interest were labeled and highlighted with arrows indicating the direction of relative expression enrichment (Control vs. DS). Plots were produced for individual cell types as well as for the aggregated dataset.

#### 2.9.4. Gene Set Expression Trend Analysis

To assess expression trends within defined gene sets, horizontal bar plots were generated showing the mean Log2FC ± standard deviation for each gene group and cell type. Bar direction indicated the directionality of expression changes (positive = DS-enriched; negative = Control-enriched).

#### 2.9.5. Computational Environment

All analyses and visualizations were performed using R (R Core Team), employing standard packages for data manipulation and plotting, which include tidyverse, ggplot2, and EnhancedVolcano.

### 2.10. Statistical Analysis

We assessed data distributions using the Shapiro–Wilk test for normality. Depending on the outcome, we conducted the comparisons using either unpaired *t*-tests, one-way ANOVA with Tukey’s post hoc test, or Kruskal–Wallis tests followed by Dunn’s post hoc correction. We carried out all statistical computations using GraphPad Prism version 10 (GraphPad Software, San Diego, CA, USA). Results with *p*-values less than 0.05 were considered statistically significant. Data are presented as mean ± standard error of the mean (SEM).

MEA: We presented all electrophysiological features herein as median ± inter-quartile range for each well, aggregating electrode information within wells and across all wells within a group. We implemented Kruskal–Wallis with post hoc Bonferroni correction in Python (v. 3.12.0, Python Software Foundation, Wilmington, DE, USA) using the open-source SciPy stats module. Significance is denoted as *p* < 0.05 (*).

## 3. Results

### 3.1. Expression of Thyroid Hormones Signaling Genes in the Cortical Brain of DS Subjects

**Figure 1:**
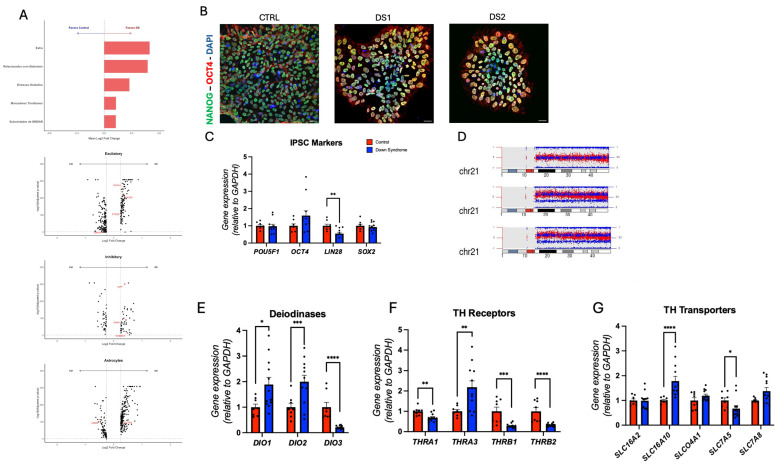
Expression of thyroid hormones signaling genes is altered in the prefrontal cortex and iPSC of individuals with Down syndrome: (**A**) single-nucleus RNA-seq data [39] from human cortex and gene expression profiling; (**B**) representative immunohistochemistry images of hiPSC. NANOG^+^, OCT4^+^ cells. Scale bar 20 μm; (**C**) relative gene expression (RT-qPCR) of *POU5F1*, *OCT4*, *LIN28*, and *SOX2*. N = 8 (control), 12 (DS) replicates; (**D**) digital karyotyping of chromosome 21; (**E**) relative gene expression (RT-qPCR) of deiodinase *DIO1*, *DIO2*, and *DIO3*. N = 8 (control), 12 (DS) replicates; (**F**) relative gene expression (RT-qPCR) of THRs *THRA1*, *THRA3*, *THRB1*, and *THRB2*. N = 8 (control), 12 (DS) replicates; (**G**) relative gene expression (RT-qPCR) of THs transporters *SLC16A2*, *SLC16A10*, *SLCO4A1*, *SLC7A5*, and *SLC7A8*. N = 8 (control), 12 (DS) replicates. * *p* < 0.05, ** *p* < 0.01, *** *p* < 0.001, **** *p* < 0.0001.

We analyzed publicly available single-nucleus RNA sequencing (snRNA-seq) data from Palmer et al. [39], which profiled the Brodmann area 8/9 of the prefrontal cortex from 29 individuals, including 20 controls and 9 with Down syndrome (DS). To align with the focus of this study, we examined the reported expression patterns of genes related to Alzheimer’s disease, oxidative stress, thyroid hormones signaling, and NMDA receptor subunit (Figure 1A). The dataset identified eight major brain cell types, several of which showed differential expression of genes of interest: excitatory neurons (*NRGN*, *PTGS2*, *NCOA1*, *BACE2*), inhibitory neurons (*HOMER1*, *GRIN1*, *APP*), astrocytes (*CIRBP*, *SLC7A5*), microglia (*SLC7A5*, *APP*), oligodendrocyte precursor cells (*NCOA1*), oligodendrocytes (*NRGN*, *CIRBP*), pericytes (*BACE2*, *APP*, *GRIN2B*), and endothelial cells (*SLC7A8*, *THRB*, *GRIN2A*, *NCOR2*, *APP*, *SLCO4A1*, *BACE2*) (Figure 1A; Supplementary Figure S1). Based on these observations, we selected representative genes for target validation and mechanistic analysis in human iPSC-derived neural cells to examine how trisomy 21 affects THs homeostasis in a neural in vitro cell model.

### 3.2. Altered Thyroid Hormones Maintenance in Down Syndrome hiPSCs

The hiPSCs were generated from fibroblasts of two individuals with Down syndrome (DS) [38] and compared to a well-characterized control hiPSC line [35,36]. We confirmed pluripotency via immunofluorescence staining for NANOG, OCT4, LIN28, and SOX2 (Figure 1B; Supplementary Figure S2A), and by RT-qPCR analysis of *POU5F1*, *OCT4*, *LIN28*, and *SOX2* gene expression (Figure 1C; Supplementary Figure S2B). *LIN28* expression, a gene implicated in cellular proliferation [40], was markedly reduced in DS-derived hiPSCs. Digital karyotyping confirmed trisomy 21 in the DS-derived hiPSCs (Figure 1D; Supplementary Figure S2C).

Gene expression analysis revealed dysregulation of THs’ regulatory pathways in DS-derived hiPSCs. The gene expression of THs activating deiodinases *DIO1* and *DIO2* was significantly upregulated, while the gene expression of THs inactivating deiodinase *DIO3* was downregulated (Figure 1E; Supplementary Figure S2D), suggesting a potential elevation of the intracellular active T3 levels in DS-derived cells. Despite this, the gene expression of the THRs *THRA1*, *THRB1*, and *THRB2* was downregulated, whereas gene expression of *THRA3* was upregulated (Figure 1F; Supplementary Figure S2E). Additionally, the gene expression of the TH transporter *SLC16A10* (MCT10) was increased, while *SLC7A5* (LAT1) was decreased (Figure 1G; Supplementary Figure S2F), indicating disrupted TH uptake and signaling in DS-derived hiPSCs.

### 3.3. Altered Thyroid Hormones Maintenance in Down Syndrome Hid-NPCs

**Figure 2:**
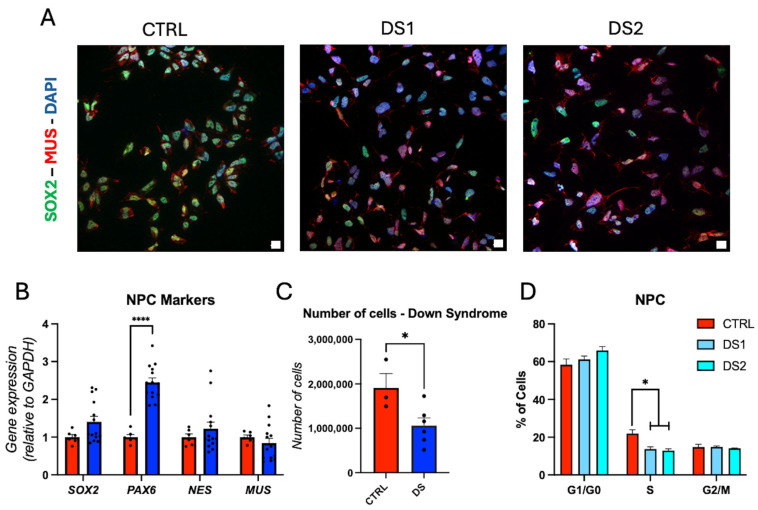
Down syndrome hid-NPCs exhibit altered cell proliferation: (**A**) representative immunohistochemistry images of hid-NPC. SOX2^+^, MUS^+^ cells. Scale bar 50 μm; (**B**) relative gene expression (RT-qPCR) of *SOX2*, *PAX6*, *NES*, and *MUS*. N = 6 (control), 14 (DS) replicates; (**C**) live cell counts for control and DS hid-NPCs. N = 3 control, N = 3 DS1, N = 3 DS2 replicates; (**D**) DS hid-NPCs show an altered cell-cycle profile compared to control. N = 3 (control) and 3 (DS) replicates. * *p* < 0.05, **** *p* < 0.0001.

**Figure 3:**
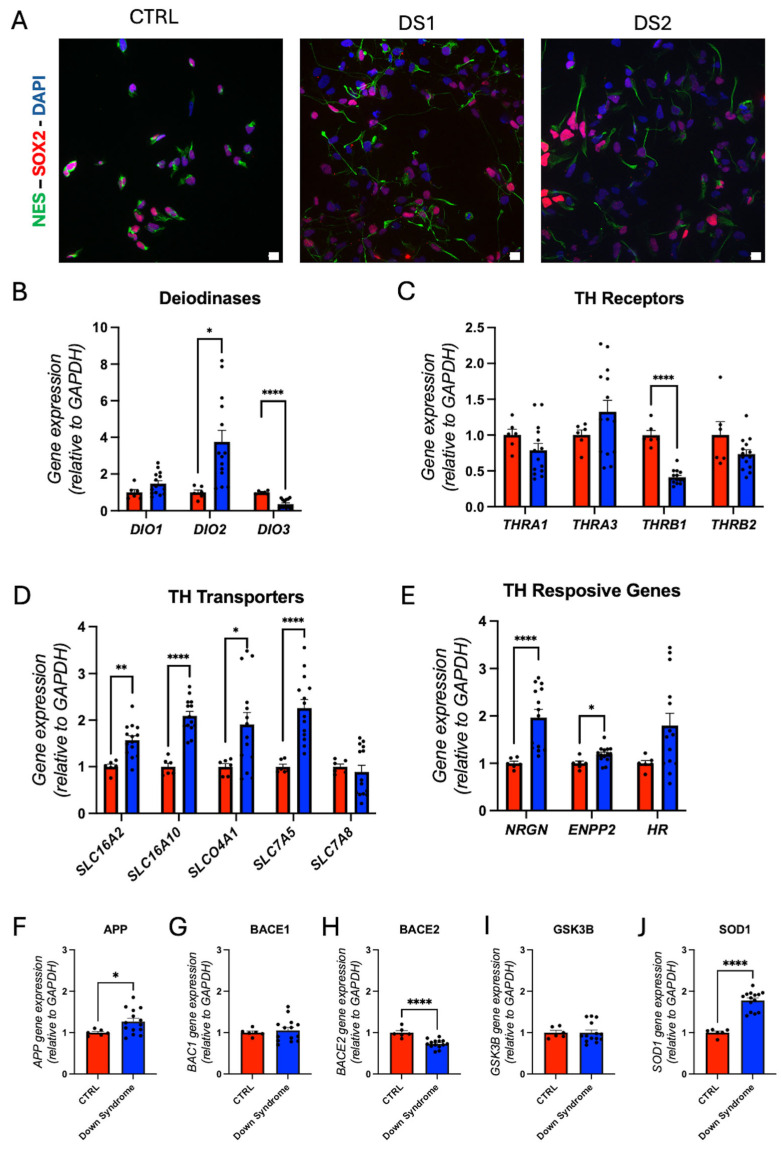
Down syndrome hiPSC-derived NPCs exhibit altered thyroid hormones signaling and dysregulation of expression of Alzheimer’s-related genes: (**A**) representative immunohistochemistry images of hid-NPC. NES^+^, SOX2^+^ cells. Scale bar 50 μm; (**B**) relative gene expression (RT-qPCR) of deiodinases *DIO1*, *DIO2*, and *DIO3*. N = 6 (control), 12 (DS) replicates; (**C**) relative gene expression (RT-qPCR) of THRs *THRA1*, *THRA3*, *THRB1*, and *THRB2*. N = 6 (control), 12 (DS) replicates; (**D**) relative gene expression (RT-qPCR) of THs transporters *SLC16A2*, *SLC16A10*, *SLCO4A1*, *SLC7A5*, and *SLC7A8*. N = 6 (control), 14 (DS) replicates; (**E**) relative gene expression (RT-qPCR) of THs responsive genes *NRGN*, *ENPP2*, and *HR*. N = 6 (control), 12 (DS) replicates; (**F–J**) relative gene expression (RT-qPCR) of Alzheimer’s-related genes *APP*, *BACE1*, *BACE2*, *GSK3B*, and *SOD1*. N = 6 (control), 12 (DS) replicates. * *p* < 0.05, ** *p* < 0.01, **** *p* < 0.0001.

We derived hid-NPCs from previously characterized hiPSCs. Immunofluorescence for SOX2, MUSASHI (MUS), and Nestin (Figure 2A and Figure 3A), along with RT-qPCR for *SOX2*, *PAX6*, *NES*, and *MUS* (Figure 2B), confirmed neuroprogenitor cell identity. *PAX6* gene expression is elevated in DS hid-NPCs. Proliferation and cell cycle analyses demonstrated reduced cell proliferation and a lower percentage of cells in the S phase (Figure 2C,D; Supplementary Figure S3A,B), indicating a decreased self-renewal capacity in the DS-derived cells. The proliferation and cell cycle findings, together with the increased expression of *PAX6*, suggest a shift toward neurogenic differentiation [41].

TH-related gene analysis showed increased *DIO2* and decreased *DIO3* gene expression (Figure 3B; Supplementary Figure S3C,D), suggesting a potential elevation in intracellular T3 levels. The THR gene *THRB1* was downregulated (Figure 3C; Supplementary Figure S3J–M). Gene expression of the THs transporter *SLC16A2* (MCT8), *SLC16A10* (MCT10), *SLCO4A1* (OATP4A1), and *SLC7A5* (LAT1) was upregulated (Figure 3D; Supplementary Figure S3E–I), reflecting enhanced THs transport. The gene expression of TH-responsive *NRGN* and *ENPP2* was also upregulated (Figure 3E; Supplementary Figure S3N–P), suggesting increased TH signaling output.

Given the location of *APP* on chromosome 21, we evaluated the expression of genes associated with Alzheimer’s disease. *APP* gene expression is upregulated in DS hid-NPCs (Figure 3F). However, *BACE1* (Figure 3G) and *GSK3B* (Figure 3I) gene expression remained unchanged, while *BACE2* (Figure 3H) gene expression was downregulated. *SOD1* (Figure 3J) is another gene present on chromosome 21, and its gene expression is upregulated in DS hid-NPC.

### 3.4. Altered Thyroid Hormones Maintenance in Down Syndrome Hid-Astrocytes

**Figure 4:**
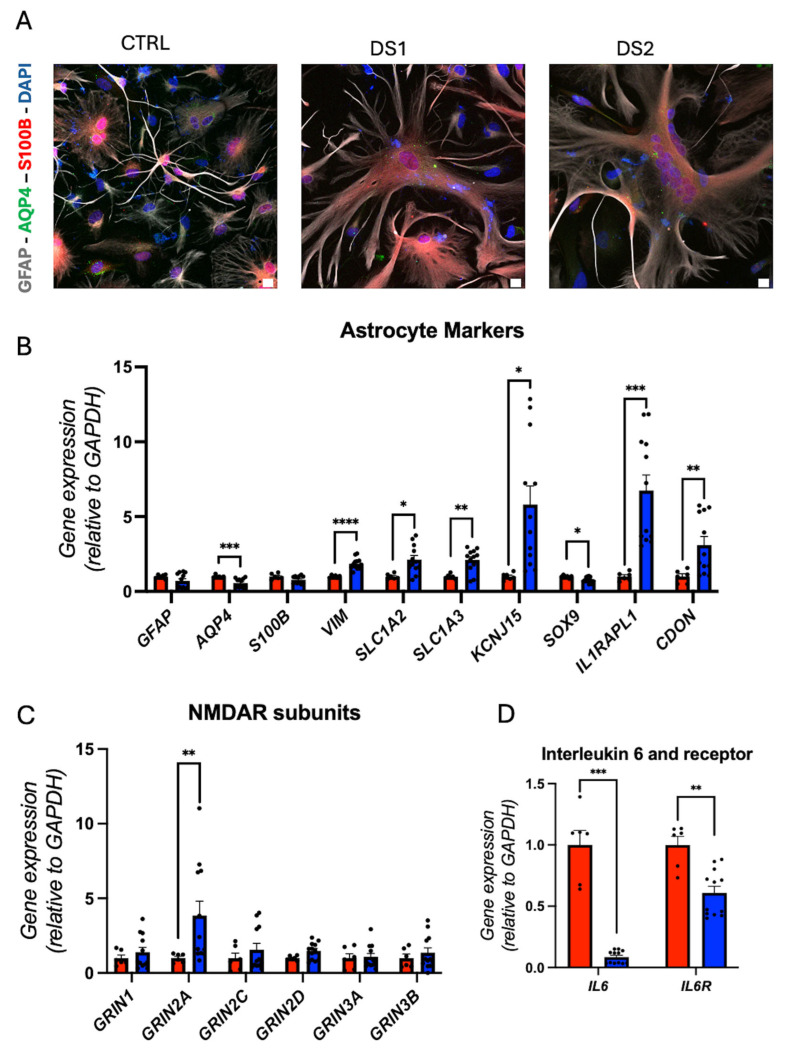
Down syndrome hid-Astrocytes display altered identity and altered glutamate homeostasis: (**A**) representative immunohistochemistry images of hid-Astrocytes. GFAP^+^, AQP4^+^, and S100B^+^ cells. Scale bar 5 μm; (**B**) relative gene expression (RT-qPCR) of astrocyte markers *GFAP*, *AQP4*, *S100B*, *VIM*, *SLC1A2*, *SLC1A3*, *KCNJ15*, *SOX9*, *IL1RAPL1*, and *CDON*. N = 6 (control), 12 (DS) replicates; (**C**) relative gene expression (RT-qPCR) of NMDA receptors subunits *GRIN1*, *GRIN2A*, *GRIN2C*, *GRIN2D*, *GRIN3A*, and *GRIN3B*. N = 6 (control), 12 (DS) replicates; (**D**) relative gene expression (RT-qPCR) of interleukin 6 and receptor *IL6*, and *IL6R*. N = 6 (control), 12 (DS) replicates. * *p* < 0.05, ** *p* < 0.01, *** *p* < 0.001, **** *p* < 0.0001.

**Figure 5:**
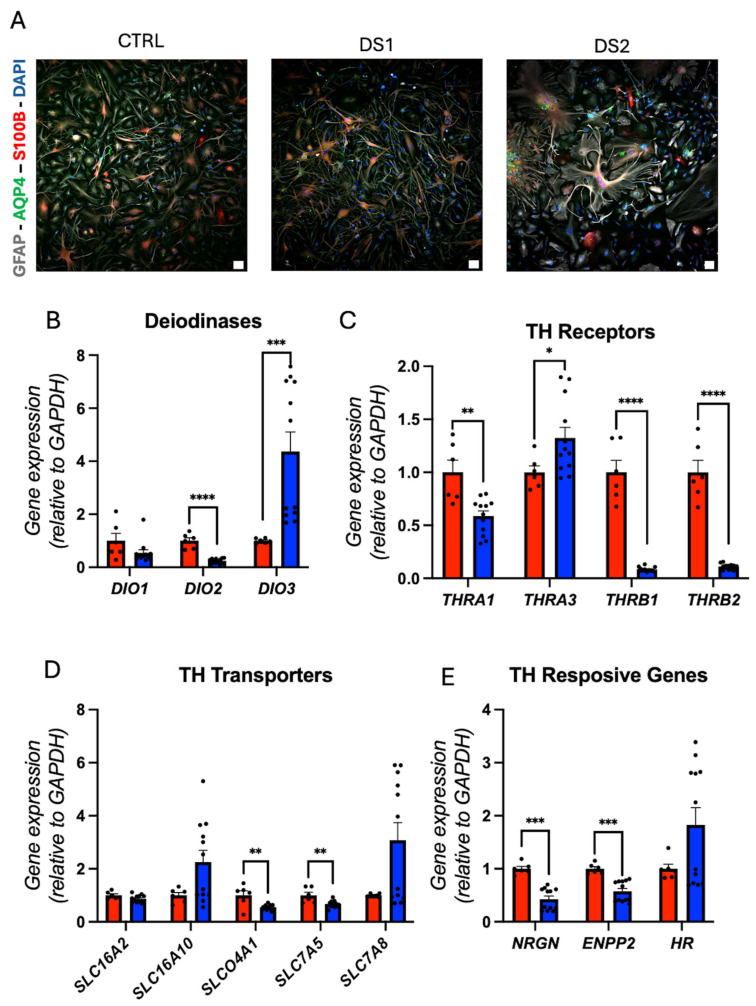
Down syndrome hid-Astrocytes present altered Thyroid hormones signaling gene expression: (**A**) representative immunohistochemistry images of hid-Astrocytes (ref. Figure 4A). GFAP^+^, AQP4^+^, and S100B^+^ cells. Scale bar 100 μm; (**B**) relative gene expression (RT-qPCR) of deiodinases *DIO1*, *DIO2*, and *DIO3*. N = 6 (control), 12 (DS) replicates; (**C**) relative gene expression (RT-qPCR) of THRs *THRA1*, *THRA3*, *THRB1*, and *THRB2* N = 6 (control), 12 (DS) replicates; (**D**) relative gene expression (RT-qPCR) of THs transporters *SLC16A2*, *SLC16A10*, *SLCO4A1*, *SLC7A5*, and *SLC7A8*. N = 6 (control), 12 (DS) replicates (**E**) relative gene expression (RT-qPCR) of THs responsive genes *NRGN*, *ENPP2*, and *HR*. N = 6 (control), 12 (DS) replicates. * *p* < 0.05, ** *p* < 0.01, *** *p* < 0.001, **** *p* < 0.0001.

We derived hid-Astrocytes from hid-NPCs and validated them by immunofluorescence staining for GFAP, AQP4, and S100B (Figure 4A and Figure 5A), and by RT-qPCR for astrocyte markers including *GFAP*, *AQP4*, *S100B*, *VIM*, *SLC1A2*, *SLC1A3*, *KCNJ15*, *SOX9*, *IL1RAPL1*, and *CDON* gene expression (Figure 4B). *AQP4* and *SOX9* gene expression was downregulated, while *VIM*, *SLC1A2*, *SLC1A3*, *KCNJ15*, *IL1RAPL1*, and *CDON* gene expression was upregulated in DS hid-Astrocytes, suggesting altered astrocytic identity and reactivity.

The concurrent upregulation of the gene expression of *SLC1A2* and *SLC1A3* (Figure 4B) with *GRIN2A* (Figure 4C) in DS hid-Astrocytes suggests a compensatory astrocytic response to a possible increased glutamatergic activity. *SLC1A2* and *SLC1A3* encode high-affinity glutamate transporters that are critical for clearing extracellular glutamate and preventing excitotoxicity [42]. This expression profile may reflect early reactive gliosis or astrocyte hyper-responsiveness, potentially triggered by elevated synaptic glutamate or disrupted neuron–astrocyte signaling in DS [38]. Trisomy 21 promotes a dysfunctional excitatory environment, as previously demonstrated by Chen and collaborators [43]. Our results showed that DS hid-Astrocytes upregulate glutamate transporters and glutamate receptor subunits, thereby altering glutamatergic homeostasis and contributing to excitatory imbalance.

Gene expression analysis revealed the downregulation of *IL6* and *IL6R* (Figure 4D), indicating altered glutamatergic signaling. This gene expression dysregulation is associated with neuronal disorders [44], and the downregulation of *IL6/IL6R* may be involved in inhibiting cell growth [45], which may be reinforced by the decreased gene expression of *SOX9*, also involved in cell proliferation [46,47].

In this context, decreased gene expression of *IL6/IL6R* may impair anti-excitotoxic feedback, potentially exacerbating glutamate accumulation or altered synaptic tone. This profile may reflect a dysfunctional astrocyte state, where glutamate handling is upregulated independently of proper cytokine-mediated regulation, contributing to excitatory-inhibitory imbalance in the DS neural environment, which Palmer et al. already confirmed in their work [39].

Next, THs regulatory pathways were also disrupted. *DIO2* gene expression was downregulated and *DIO3* gene expression was upregulated (Figure 5B), suggesting a potential decrease in the intracellular T3 availability. *THRA1*, *THRB1*, and *THRB2* gene expression were all downregulated, and *THRA3* gene expression was upregulated (Figure 5C). Gene expression of the THs transporter *SLCO4A1* (OATP4A1) and *SLC7A5* (LAT1) was decreased (Figure 5D). Correspondingly, the gene expression of *NRGN* and *ENPP2*, both TH-responsive genes, was downregulated (Figure 5E), confirming reduced TH action.

### 3.5. Altered Thyroid Hormones Maintenance in Down Syndrome Hid-Neurons

**Figure 6:**
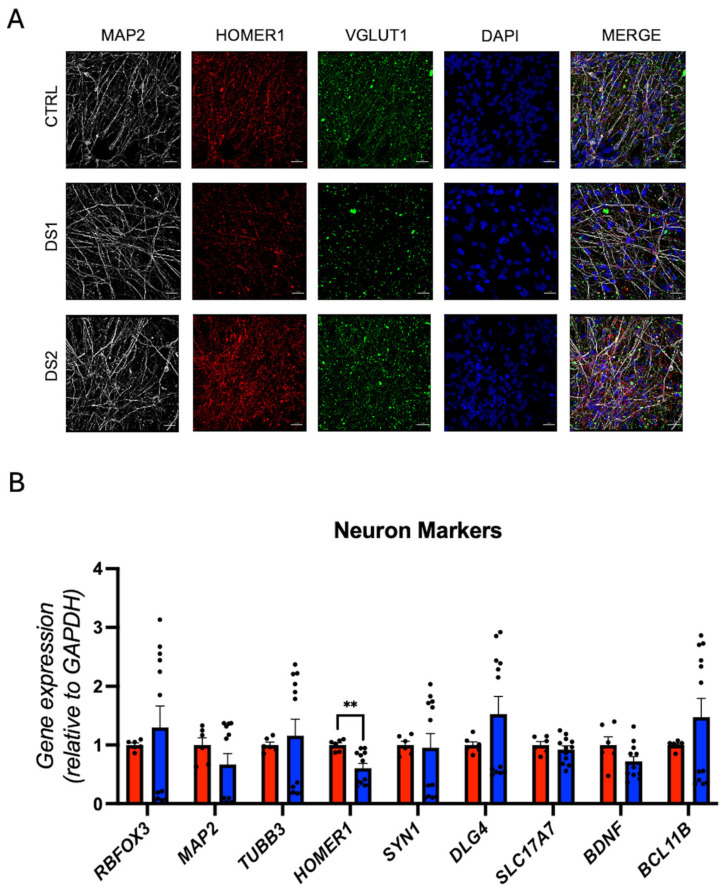
Down syndrome hiPSC-derived neurons exhibit synaptic gene expression dysregulation: (**A**) representative immunohistochemistry images of neurons. MAP2^+^, HOMER1^+^, and VGLUT1^+^ cells. Scale bar 20 μm; (**B**) relative gene expression (RT-qPCR) of neuron markers *RBFOX3*, *MAP2*, *TUBB3*, *HOMER1*, *SYN1*, *DLG4*, *SLC17A7*, *BDNF*, and *CTIP2*. N = 6 (control), 12 (DS) replicates. ** *p* < 0.01.

We derived the hid-Neurons from DS and control hid-NPCs and validated them by immunostaining for MAP2, HOMER1, VGLUT1, NEUN, and CTIP2 (Figure 6A; Figure 8A). RT-qPCR confirmed gene expression of neuronal markers *RBFOX3* (NEUN), *MAP2*, *TUBB3*, *HOMER1*, *SYN1*, *DLG4* (PSD95), *SLC17A7* (VGLUT1), *BDNF*, and *CTIP2* (Figure 6B; Supplementary Figure S4A–H). Notably, *HOMER1*, a synaptic scaffolding gene [48], was downregulated in DS hid-Neurons.

**Figure 7:**
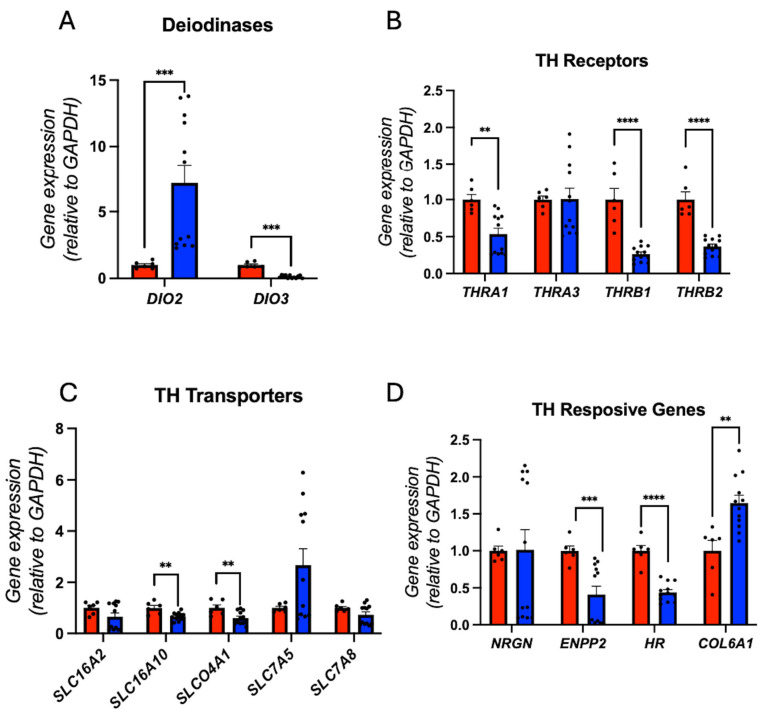
Down syndrome hid-Neurons presented altered Thyroid hormones signaling: (**A**) relative gene expression (RT-qPCR) of deiodinases DIO2 and DIO3. N = 6 (control), 12 (DS) replicates; (**B**) relative gene expression (RT-qPCR) of THRs THRA1, THRA3, THRB1, and THRB2. N = 6 (control), 12 (DS) replicates; (**C**) relative gene expression (RT-qPCR) of THs transporters SLC16A2, SLC16A10, SLCO4A1, SLC7A5, and SLC7A8. N = 6 (control), 12 (DS) replicates; (**D**) relative gene expression (RT-qPCR) of THs responsive genes NRGN, ENPP2, HR, and COL6A1. N = 6 (control), 12 (DS) replicates. ** *p* < 0.01, *** *p* < 0.001, **** *p* < 0.0001.

As in other cell types, hiPSC, hid-NPC (earlier stages before neuron differentiation), and hid-Astrocyte THs metabolism was dysregulated: the gene expression of *DIO2* was increased, and *DIO3* was decreased (Figure 7A; Supplementary Figure S5A,B), suggesting a potential elevation in intracellular T3 levels. Despite this, the gene expression of *THRA1*, *THRB1*, and *THRB2* was downregulated (Figure 7B; Supplementary Figure S5C–F), while the gene expression of *SLC16A10* (MCT10) and *SLCO4A1* (OATP4A1) was downregulated (Figure 7C; Supplementary Figure S5G–K). The gene expression of TH-responsive genes *ENPP2* and *HR* was downregulated, while *COL6A1* was upregulated (Figure 7D; Supplementary Figure S5L–O), reflecting impaired receptor-mediated THs action.

**Figure 8:**
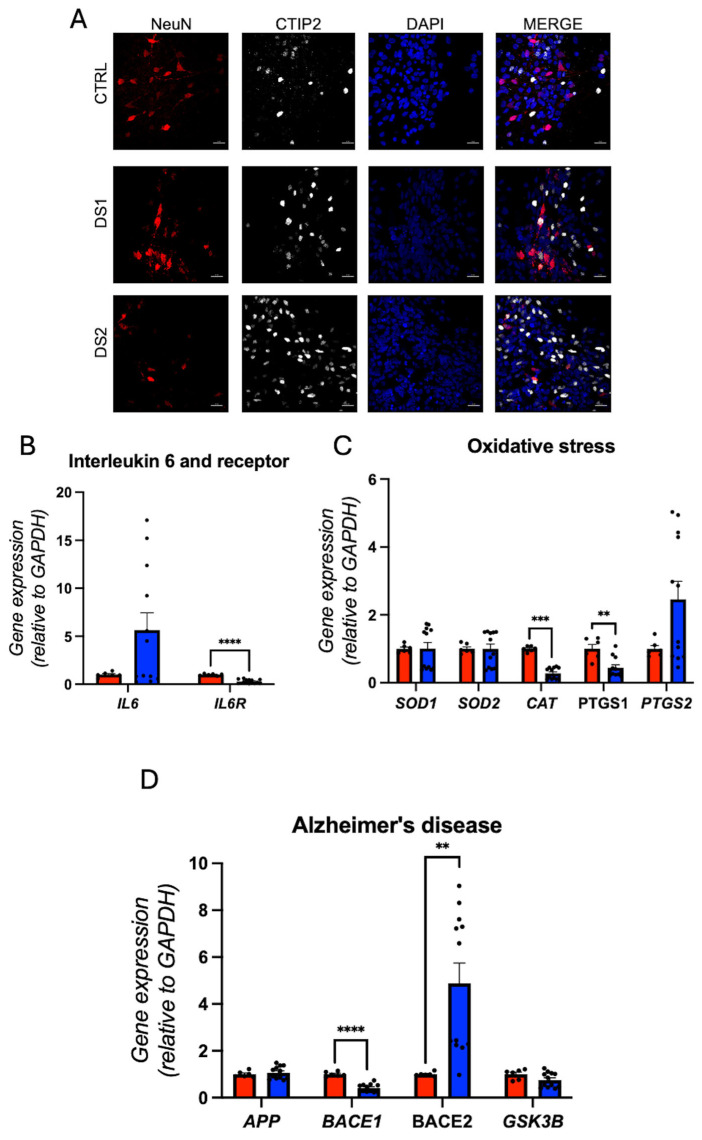
Down syndrome hid-Neurons exhibit dysregulation of the expression of Alzheimer’s-related genes: (**A**) representative immunohistochemistry images of hid-Neurons. NeuN^+^ and CTIP2^+^ cells. Scale bar 20 μm; (**B**) relative gene expression (RT-qPCR) of *IL6* and *IL6R*. N = 6 (control), 12 (DS) replicates; (**C**) relative gene expression (RT-qPCR) of oxidative stress genes *SOD1*, *SOD2*, *CAT*, *PTGS1*, and *PTGS2*. N = 6 (control), 12 (DS) replicates; (**D**) relative gene expression (RT-qPCR) of Alzheimer’s-related genes *APP*, *BACE1*, *BACE2*, and *GSK3B*. N = 6 (control), 12 (DS) replicates. ** *p* < 0.01, *** *p* < 0.001, **** *p* < 0.0001.

Pro-inflammatory and oxidative stress markers gene expression was altered, *IL6AR* was decreased (Figure 8B; Supplementary Figure S6A,B), and *CAT* and *PTGS1* gene expression was dysregulated (Figure 8C; Supplementary Figure S6C–G). Alzheimer’s-related gene expression showed reduced *BACE1* and increased *BACE2* (Figure 8D; Supplementary Figure S6H–K).

### 3.6. NMDAR Subunits Gene Expression and Network Activity in Down Syndrome Hid-Neurons Are Altered

**Figure 9:**
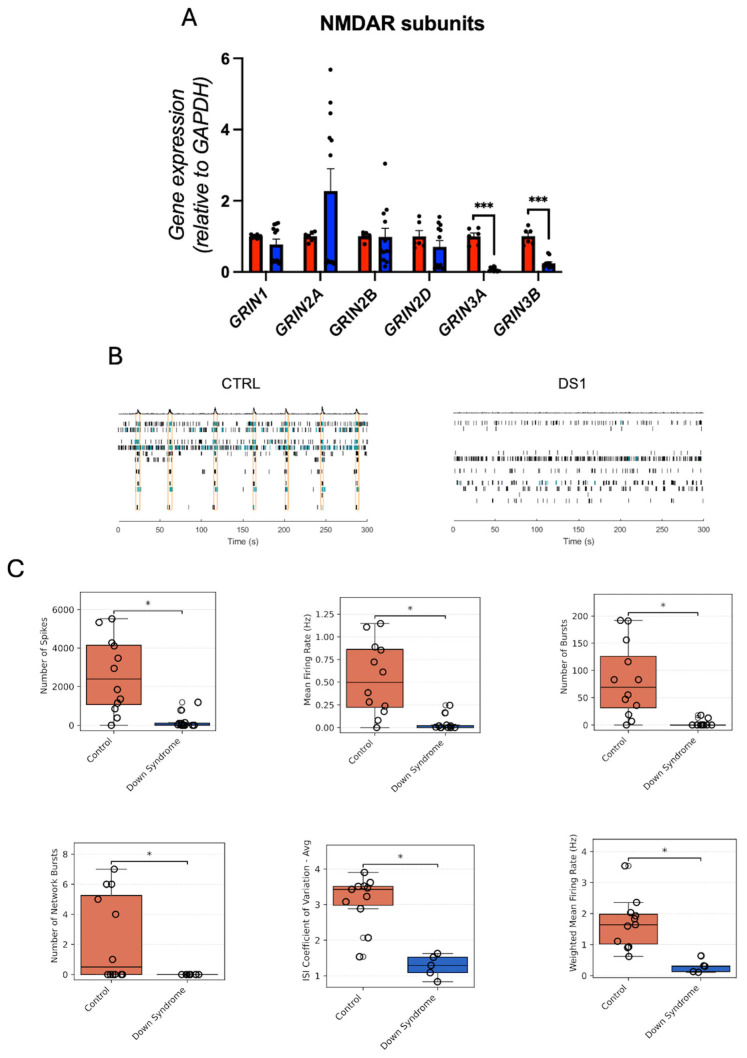
Down syndrome hid-Neurons exhibit deficient network activity: (**A**) relative gene expression (RT-qPCR) of NMDA receptor subunits *GRIN1*, *GRIN2A*, *GRIN2C*, *GRIN2D*, *GRIN3A*, and *GRIN3B*. N = 6 (control), 12 (DS) replicates; (**B**) representative MEA raster plots for control and DS hid-Neurons. Yellow rectangles were generated by Axion NeuralMetric software and denote network bursts. (**C**) Comparison of control and DS hid-Neurons of the number of spikes, mean firing rate, number of bursts, number of network bursts, ISI coefficient of variation, and weighted mean firing rate. N = 12. * *p* < 0.05; *** *p* < 0.001.

NMDA receptor subunit gene expression profiling revealed significant downregulation of *GRIN3A* and *GRIN3B* in DS hid-Neurons (Figure 9A; Supplementary Figure S7A–F), suggesting disruption in synaptic NMDA receptor composition and function.

To assess the functional consequences, we conducted MEA recordings on DS and control hid-Neurons cultured in 2D conditions. DS hid-Neurons displayed a significantly decreased number of spikes, mean fire rate, number of bursts, number of network bursts, inter-spike interval (ISI) coefficient of variance, and reduced weighted mean firing rate (Figure 9B,C; Supplementary Figure S7G) compared to control. Treatment with T3 or GC1 did not rescue these deficits, suggesting that altered THRs gene expression and transport may limit responsiveness to exogenous T3 and GC1.

## 4. Discussion

This study demonstrates that THs maintenance is profoundly altered across multiple stages of neural differentiation in human iPSC-derived cells from individuals with DS. Consistent with prior suggestions of endocrine-metabolic disruption in DS [27,49], our data reveal the expression of deiodinases, transporters, and receptors, as well as TH-responsive genes. These molecular changes coincide with reduced hid-NPC proliferation, disrupted synaptic gene expression, and impaired network activity, supporting a cell-intrinsic contribution to neural dysfunction in trisomy 21.

Both hiPSCs and hid-NPCs from DS exhibit increased gene expression of activating deiodinases (*DIO1* and *DIO2*) and decreased gene expression of *DIO3*, suggesting elevated intracellular T3 production. Despite this, THRs transcripts (*THRA1*, *THRB1*, *THRB2*) were reduced, yet transcriptional activation of canonical targets (*NRGN* and *ENPP2*) persisted. These findings extend prior reports that TH-responsive genes may be regulated through non-genomic or receptor-independent mechanisms, particularly under disrupted intracellular THs balance [50,51].

Our hid-NPC data also showed increased *PAX6* expression, a key neurogenic factor, alongside reduced proliferation. This aligns with previous findings that THs promote neurogenic over proliferative division [52,53], suggesting that THs may accelerate premature neural differentiation. Such a shift could deplete the progenitor pool prematurely, as suggested in prior models of accelerated neurogenesis in DS.

In contrast, hid-Astrocytes derived from DS hid-NPCs exhibited decreased gene expression of *DIO2*, THs transporters (*SLC7A5*, *SLCO4A1*), and THRs, along with downregulation of the expression of TH-responsive genes (*NRGN*, *ENPP2*). This pattern suggests a localized state of hypothyroidism in astrocytes, consistent with observations in murine and human studies that THs signaling in glia is essential for maintaining synaptic and metabolic homeostasis [54]. Our findings underscore the cell-type specificity of THs impairment in DS, which may be obscured in systemic evaluations of thyroid function.

Neurons exhibited a mixed profile, with *DIO2* gene expression elevated and *DIO3* gene expression decreased, while both transporter and receptor genes were downregulated, and TH-response genes showed inconsistent gene expression regulation. We interpret this as a possible compensatory response to the limited astrocyte-derived T3, consistent with the known paracrine role of astrocytes in supplying T3 to neurons [55]. Moreover, the downregulation of gene expression of *HOMER1*, *GRIN3A*, and *GRIN3B* suggests impaired excitatory synaptic structure and signaling. HOMER1 is a scaffold for mGluR-NMDAR complexes critical for synaptic plasticity [56], and *GRIN3A* and *GRIN3B* encode GluN3 subunits that modulate NMDAR channel properties [57]. Together, these changes may sensitize DS neurons to excitotoxicity and weaken synaptic integration.

Supporting these molecular findings, MEA recordings revealed reduced spontaneous firing, bursting, and synchrony in DS hid-Neurons. These electrophysiological deficits parallel the molecular evidence of diminished excitatory signaling, reinforcing the functional impact of THs signaling impairment. Notably, treatment with T3 or the TRβ-selective agonist GC1 failed to rescue these impairments, consistent with previous findings of reduced THs responsivity in certain pathological contexts due to receptor or signaling machinery limitations [19].

The additional observation of altered gene expression in Alzheimer’s disease-related genes (*APP*, *BACE2*, *SOD1*) and oxidative stress markers (*CAT*, *PTGS1*) in DS hid-Neurons and hid-Astrocytes extends recent hypotheses linking early THs dysregulation with pathways relevant to DS-associated neurodegeneration [24]. These results underscore the long-term implications of impaired intracellular hormone regulation beginning in early neurodevelopment.

## 5. Limitations and Outlook

Although this study employed two DS-derived iPSC lines and one control, the sample size is in line with prior iPSC-based DS studies [38,43]. Our findings were consistent across replicates, cell types, and methodological approaches (gene expression and electrophysiology). We acknowledge that future studies incorporating a broader panel of donor lines, including protein-level validations and organoid-based models, would enhance generalizability. However, the current dataset, which integrates transcriptional and functional data, provides robust and novel insights into the cell-specific disruption of THs signaling in DS.

## 6. Conclusions

**Figure 10:**
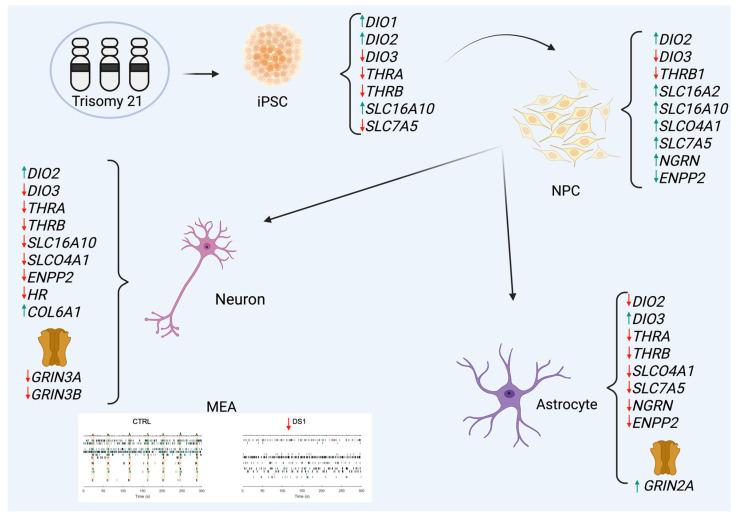
Cell-type-specific dysregulation of thyroid hormones signaling in Down syndrome hiPSC-derived neural cells. Trisomy 21 hiPSCs, hid-NPCs, hid-Neurons, and hid-Astrocytes derived from individuals with Down syndrome show distinct disruptions in THs maintenance pathways. hiPSCs and hid-NPCs have increased *DIO2* gene expression and decreased *DIO3* gene expression, along with altered expression of THs transporters (e.g., *SLC16A10*, *SLC7A5*) and receptors (*THRA*, *THRB*). In hid-NPCs, THs-responsive genes (*NRGN*, *ENPP2*) are upregulated, indicating a hyperthyroid-like state. hid-Astrocytes, on the other hand, exhibit reduced *DIO2* and increased *DIO3* gene expression, accompanied by decreased levels of gene expression of transporters and receptors, indicating localized hypothyroidism. hid-Neurons exhibit similar THs dysregulation and reduced gene expression of excitatory synaptic genes (*GRIN3A*, *GRIN3B*). Electrophysiological recordings confirm decreased neuronal activity in DS hid-Neurons. This schematic emphasizes a significant, stage-specific imbalance in THs signaling throughout hiPSC and hiPSC-derived neural cells in DS. Created in https://BioRender.com. (accessed on 27 August 2025).

In conclusion, this study reveals that trisomy 21 induces cell-type-specific disruptions in THs metabolism and signaling during human neural differentiation (Figure 10). These disruptions affect both early lineage specification and late-stage synaptic integration, likely contributing to the cognitive and neuropsychiatric manifestations of DS. Our findings highlight the importance of considering THs signaling not merely as a systemic parameter but as a dynamic, cell-autonomous process with implications for therapeutic intervention. hiPSC-based models offer a valuable platform for testing the efficacy of THs analogs and receptor modulators tailored to DS-associated vulnerabilities.

## Data Availability

Single-nucleus RNA sequencing transcriptomic data and associated metadata were obtained from Palmer et al. (EGAS00001005691) [39].

## Supplementary Materials

The following supporting information can be downloaded at https://www.mdpi.com/article/10.3390/cells14181407/s1. Supplementary Table S1: List of antibodies; Supplementary Table S2: List of primers; Supplementary Table S3: List of abbreviations; Figure S1: Down syndrome presented more cell types altered compared to the control; Figure S2: Validation of pluripotency and expanded THs gene expression profiles in DS hiPSC; Figure S3: Extended analysis of cell cycle, THs pathway, and AD-related gene expression in DS hid-NPCs; Figure S4: Extended analysis of hid-Neuron markers gene expression; Figure S5: Extended analysis of gene expression of the THs pathway in DS hid-Neurons; Figure S6: Extended analysis of gene expression of Alzheimer’s-related genes; Figure S7: Extended analysis of synaptic deficits in Down syndrome hid-Neurons.
