# Supplementary material for: Trisomy 21 Disrupts Thyroid Hormones Signaling During Human iPSC-Derived Neural Differentiation In Vitro
Source: Cells. 2025 Sep 9;14(18):1407. doi: 10.3390/cells14181407 (PMC12468339; doi:10.3390/cells14181407)
Supplement: Supplementary file 1 [file cells-14-01407-s001.zip › cells-3812362-supplementary.pdf]

Article – Supplementary files

Trisomy 21 disrupts thyroid hormones signaling during human iPSC-derived neural differentiation in vitro

Janaina Sena de Souza[1*], Sandra Sanchez-Sanchez[1], Nicolas Amelinez-Robles[2], B. S. Guerra[1], Gisele Giannocco[3,4,5] , Alysson R. Muotri[1]

Supplementary Tables.

Supplementary Table S1. List of antibodies.

ANTIBODIES	SOURCE	IDENTIFIER	CONCENTRATION
**GOAT ANTI-NANOG**	R&D	AF1997	1:500
**RABBIT ANTI-OCT4**	Abcam	Ab19857	1:500
**RABBIT ANTI-LIN28A**	Cell Signaling	3978	1:500
**RABBIT ANTI-SOX2**	Cell Signaling	2748	1:500
**GOAT ANTI-SOX2**	R&D systems	AF2018	1:250
**RABBIT ANTI-MUSASHI**	Abcam	Ab52865	1:500
**MOUSE ANTI-NESTIN**	Abcam	Ab22035	1:500
**CHICKEN ANTI-GFAP**	Abcam	ab4674	1:1000
**MOUSE ANTI-AQP4**	Abcam	ab11026	1:500
**RABBIT ANTI-S100B**	Abcam	ab52642	1:500
**CHICKEN ANTI-MAP2**	Fisher Scientific	01670262	1:1000
**RABBIT - HOMER1**	Synaptic Systems	160003	1:500
**MOUSE ANTI-VGLUT1**	Synaptic Systems	135311	1:500
**MOUSE ANTI-NEUN**	Millipore	MAB377	1:500
**RAT ANTI-CTIP2**	Abcam	Ab18465	1:500

Supplementary Table S2. List of primers.

GENE ID	FORWARD	REVERSE	SOURCE
POU5F1	TACCTCAGCCTCCAGCAGAT	ACCAGGTCTTCACCTGTTTGT	IDT
OCT4	CTCTTTTGACTGGCCTCCCC	GGGTTTCTGCTTTGCATATCTCC	IDT
LIN28	GCGGGCATCTGTAAGTGGTT	TGTGCAGCTTACTCTGGTGC	IDT
SOX2	CATGAAGGAGCACCCGGATT	TAACTGTCCATGCGCTGGTT	IDT
DIO1	CCCATTTCAGCCACGACAAC	GCACCAGTGGCCTATTACCTTG	IDT
DIO2	ATGCTGACCTCAGAGGGACT	TCCTCACCCAATTTCACCTGTT	IDT
DIO3	CCCACTGCTGATGACGAACT	ACCCTCCCCCTCAAGGTTTA	IDT
THRA1	AACACAACATTCCGCACTTCTG	ACTTTCATGTGGAGGAAGCGG	IDT
THRA3	CCAAGCTGCTGATGAAGGGTC	TGGAGACTTCCCGCTTCACC	IDT
THRB1	TGCGATTTCCTTCTGGTTGG	TTCACTGACATCTCCTTCTTATTCA	IDT
THRB2	AGGGCACTGATCTGCGATTT	TGCTGGAGTTTGCCTCTCTG	IDT
SLC16A2	CAACGCACTTACCGCATCTG	CCTCCTCCACATACTTCATCAGG	IDT
SLC16A10	GTGTCCATGCTGGAAACCTTC	GAGAACCTACCCATGCTGTCTT	IDT

SLCO4A1	CCTGTACAAGGTGCTGGGC	GGCCATCTGAAGACTCCGAC	IDT
SLC7A5	CTGGTCTTCGCCACCTACCT	TGAGCAGCAGCACGCAGA	IDT
SLC7A8	CGGAGTAGCCCTGAAGAAAGA	GCCGATGATGTTCCCTACGAT	IDT
NES	CGCACCTCAAGATGTCCCTC	CAGCTTGGGGTCCTGAAAGC	IDT
PAX6	CCATCACCAATCAGCATAGGAAT	GTGCTGCTGTTGTTGCTTGA	IDT
MUS	GTCTCGAGTCATGCCCTACG	ACACGGAATTCGGGGAACTG	IDT
NRGN	GCATTTTCAAAGTTCCCGAGG	AACACGGCAGGGAAGTCTC	IDT
ENPP2	AAGGTAGAGCCAAAGAACAAGT	TCGCCCATAGAGGAGGTGT	IDT
HR	GCTTTCTCCAGATGGTGTGC	AGCCCTGCATCCAGGTAGC	IDT
APP	TCAGGGACCAAAACCTGCAT	CTCACCAACTAAGCAGCGGT	IDT
BACE1	CCGGGAGACCGACGAAGA	ACCAGGATGTTGAGCGTCTG	IDT
BACE2	CCGCGCATCTCTGATTCCA	CGGAATGACCTGCTGGAGTT	IDT
GSK3B	TCCACCTCTGGCTACCATCC	TTAGCATCTGACGCTGCTGT	IDT
SOD1	CTGTACCAGTGCAGGTCCTC	CCAAGTCTCCAACATGCCTCT	IDT
GFAP	TGCCTATAGACAGGAAGCAGA	CCTCCTCCAGCGACTCAATC	IDT
AQP4	GGCCGTAATCTGACTCCCAG	AAGGTCCACACTTACCCCAC	IDT
S100B	GGTGAGACAAGGAAGAGGATGT	GATGAGCTCCTTCAGTTCGGA	IDT
VIM	GGCGAGGAGAGCAGGATTTC	TGGGTATCAACCAGAGGGAGT	IDT
SLC1A2	GAGGCGCTAAAGGGCTTACC	GGCATATTGTTGGCACCTTCC	IDT
SLC1A3	ACATGAAGGAACAGGGGCAG	CACGGGGGCATACCACATTA	IDT
KCNJ15	TGCAGGCAGTAGCAGAATCC	GGCTCTGGAAACACTGGTCA	IDT
SOX9	GGCAAGCTCTGGAGACTTCTG	CCCGTTCTTCACCGACTTCC	IDT
IL1RAPL1	TGACCAGCAATTACCTTACCGA	TCTTAAAGGCCGTTCCCCAC	IDT
CDON	CCCGCGGAGAGTGAACC	TTTCTGGACAGCAGAGAGCG	IDT
GRIN1	AGTGGTAGAGCAGAGCCTGA	GGGATGGTACTGCGTGTCTT	IDT
GRIN2A	GAGCCTCCGGCTGGGATA	CACTGACGGTCCCTGTAGC	IDT
GRIN2C	CTTCACAGCCCGAGTGACC	CCCTGAGCTGCTAAACACCA	IDT
GRIN2D	AGAACACAGCGAGTGTGTGA	GGCTACACATGTTGCTCGGG	IDT
GRIN3A	AAGGATACGGCATTGGCCTC	AAGGATACGGCATTGGCCTC	IDT
GRIN3B	GGGCTGCTGGCGCTG	GCCAGGAACCGTGCCAA	IDT
IL6	GGCACTGGCAGAAAACAACC	CACCAGGCAAGTCTCCTCAT	IDT
IL6R	ACGCCTTGGACAGAATCCAG	GAATCTTGCACTGGGAGGCT	IDT
RBFOX3	CCAAGCGGCTACACGTCTC	CGTCCCATTCAGCTTCTCCC	IDT
MAP2	GCTCCCGGAGAAGGATTCTG	CAAGCTGAAGAATCAGCGCA	IDT
TUBB3	ATCGGGGCCAAGTTCTGG	AAGAGATGTCCAAAGGCCCC	IDT
HOMER1	GCGGGGATCTTCAGTCTCCT	TGCTGATTGCTGAACTATGTGA	IDT
SYN1	CAGCTCAACAAATCCCAGTCTCT	GCGGATGGTCTCAGCTTTCA	IDT
DLG4	AGCCCCAGGATATGTGAACG	TCACCGATGTGTGGGTTGTC	IDT
SLC17A7	TCACTCAGCTCCCATCTCCT	CATCAGAAACGCTGGTGAGA	IDT
BDNF	TCTGGTGCAGCTGGAGTTTAT	AGGGCTTTCTTTCACCGGG	IDT
CTIP2	TAGAAGTGCGCTTTCCACCT	TTCAAACTGCTTGGCCTCTT	IDT
COL6A1	TCCAGCCCCTTCTTGATAGC	CAACCTGAGGGACAGGTACT	IDT
SOD2	AACCCAAAGGGGAGTTGCTG	GCCTGTTGTTCCTTGCAGTG	IDT
CAT	CTCCGGAACAACAGCCTTCT	ATAGAATGCCCGCACCTGAG	IDT
PTGS1	TCTTGCTGTTCCTGCTCCTG	AACAGGGATTCACTGGCGTG	IDT
PTGS2	CAAATTGCTGGCAGGGTTGC	AGGGCTTCAGCATAAAGCGT	IDT
GAPDH	TCGGAGTCAACGGATTTGGT	TGAAGGGGTCATTGATGGCA	IDT

Supplementary Table S3. List of abbreviations.

ABBREVIATION	DEFINITION
**ABM**	Astrocyte Basal Medium
**CTRL**	Control
**DIO 1, 2 AND 3**	Deiodinase 1, 2 and 3
**DORSO**	Dorsomorphin dihydrochloride
**DS**	Down syndrome
**EB**	Embryonic Body
**HIPSC**	Human induced Pluripotent Stem Cell
**MEA**	Multi-Electrode Array
**NIM**	Neural Induction Medium
**HID-ASTROCYTE**	Human iPSC-derived Astrocytes
**HID-NEURON**	Human iPSC-derived Neuron
**HID-NPC**	Human iPSC-derived Neural Progenitor Cell
**PBS**	Phosphate-Buffered Saline
**RI**	ROCK Inhibitor Y-27632 dihydrochloride
**RT**	Room temperature
**SB**	SB-431542
**SMADI**	SMAD inhibitors
**SNRNA-SEQ**	single-nucleus RNA sequencing
**T3**	Triiodothyronine
**T4**	Thyroxine
**TH**	Thyroid hormone
**THR**	Thyroid hormone receptor

Supplementary Figures.

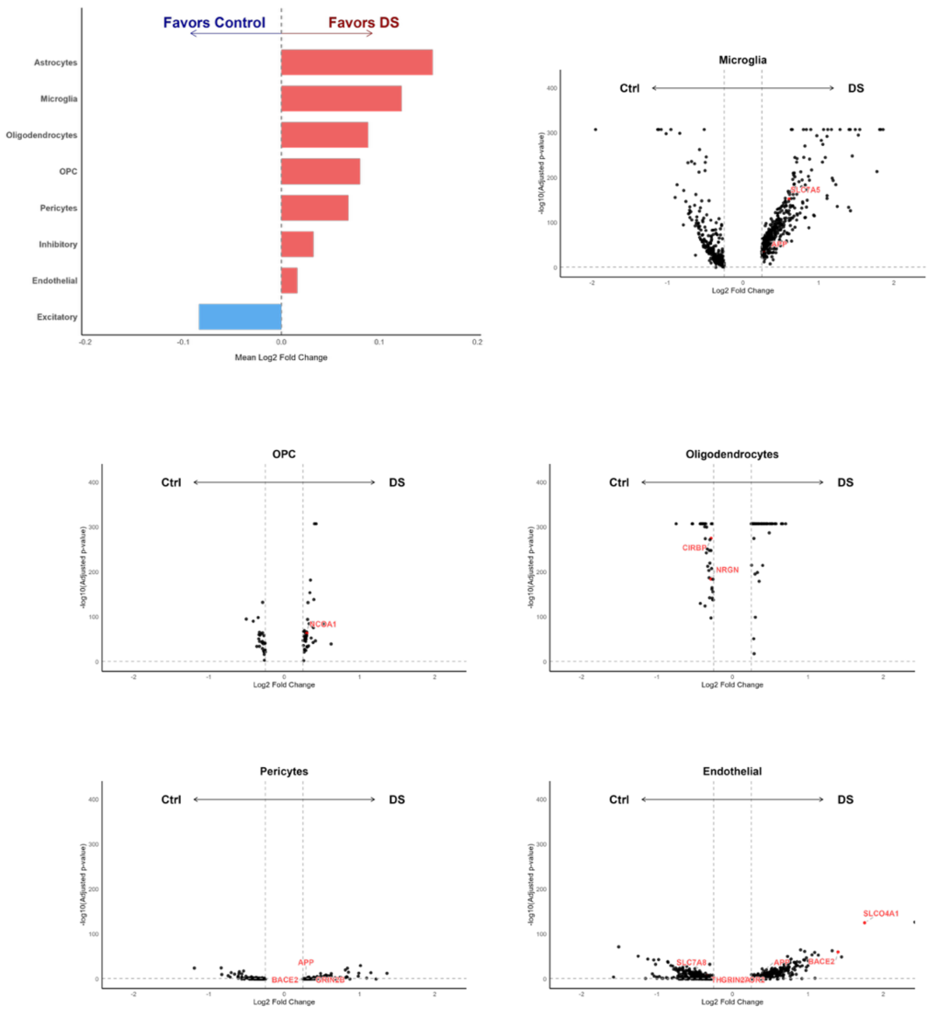

Figure S1. Down syndrome presented more cell types altered compared to control: (**a-f**) Single-nucleus RNA-seq data *[36]* from human cortex and gene expression profiling; (**b-f**) Volcano plot of microglia, OPC (oligodendrocyte progenitor cell), pericytes, and endothelial cells.

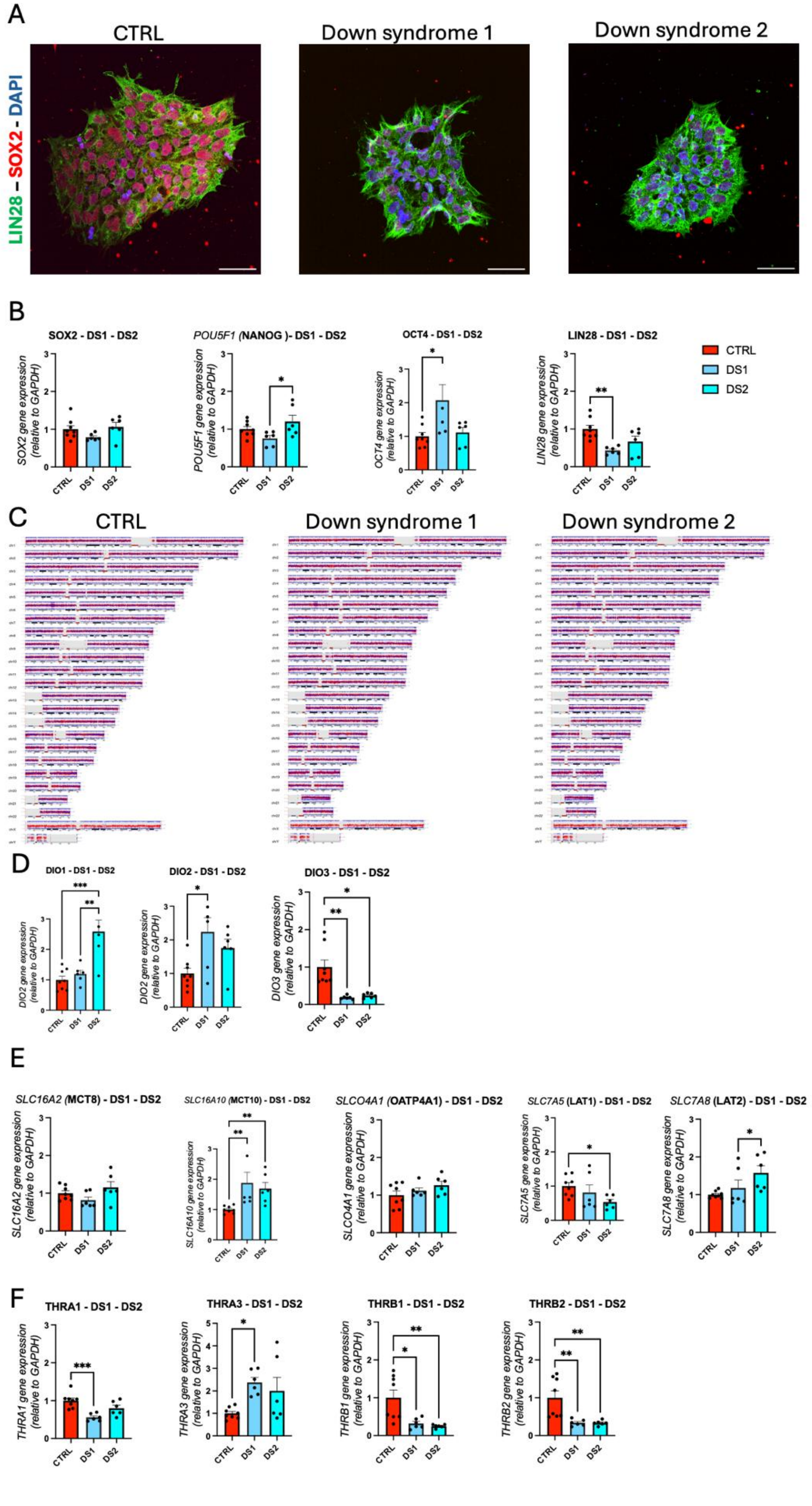
A
CTRL
Down syndrome 1
Down syndrome 2
LIN28 – SOX2 - DAPI
B
SOX2 - DS1 - DS2
POU5F1 (NANOG)- DS1 - DS2
OCT4 - DS1 - DS2
LIN28 - DS1 - DS2
CTRL
DS1
DS2
SOX2 gene expression (relative to GAPDH)
POU5F1 gene expression (relative to GAPDH)
OCT4 gene expression (relative to GAPDH)
LIN28 gene expression (relative to GAPDH)
C
CTRL
Down syndrome 1
Down syndrome 2
D
DIO1 - DS1 - DS2
DIO2 - DS1 - DS2
DIO3 - DS1 - DS2
DIO2 gene expression (relative to GAPDH)
DIO2 gene expression (relative to GAPDH)
DIO3 gene expression (relative to GAPDH)
E
SLC16A2 (MCT8) - DS1 - DS2
SLC16A10 (MCT10) - DS1 - DS2
SLCO4A1 (OATP4A1) - DS1 - DS2
SLC7A5 (LAT1) - DS1 - DS2
SLC7A8 (LAT2) - DS1 - DS2
SLC16A2 gene expression (relative to GAPDH)
SLC16A10 gene expression (relative to GAPDH)
SLCO4A1 gene expression (relative to GAPDH)
SLC7A5 gene expression (relative to GAPDH)
SLC7A8 gene expression (relative to GAPDH)
F
THRA1 - DS1 - DS2
THRA3 - DS1 - DS2
THRB1 - DS1 - DS2
THRB2 - DS1 - DS2
THRA1 gene expression (relative to GAPDH)
THRA3 gene expression (relative to GAPDH)
THRB1 gene expression (relative to GAPDH)
THRB2 gene expression (relative to GAPDH)
CTRL
DS1
DS2

Figure S2. Validation of pluripotency and expanded THs gene expression profiles in DS hiPSC: (**a**) Representative immunohistochemistry images of hiPSC. $LIN28^+$, $SOX2^+$ cells. Scale bar 20 μm; (**b**) Relative gene expression (RT-qPCR) of *POU5F1, OCT4, LIN28*, and *SOX2*. N=8 (control), 6 (DS1), and 6 (DS2) replicates; (**c**) Digital karyotyping of all chromosomes; (**d**) Relative gene expression (RT-qPCR) of deiodinase *DIO1*, *DIO2*, and *DIO3*. N=8 (control), 6 (DS1), and 6 (DS2) replicates; (**e**) Relative gene expression (RT-qPCR) of THs transporters *SLC16A2, SLC16A10, SLCO4A1, SLC7A5*, and *SLC7A8*. N=8 (control), 6 (DS1), and 6 (DS2) replicates; (**f**) Relative gene expression (RT-qPCR) of THRs *THRA1*, *THRA3*, *THRB1*, and *THRB2.* N=8 (control), 6 (DS1), and 6 (DS2) replicates. * $p < 0.05$, ** $p < 0.01$, *** $p < 0.001$.

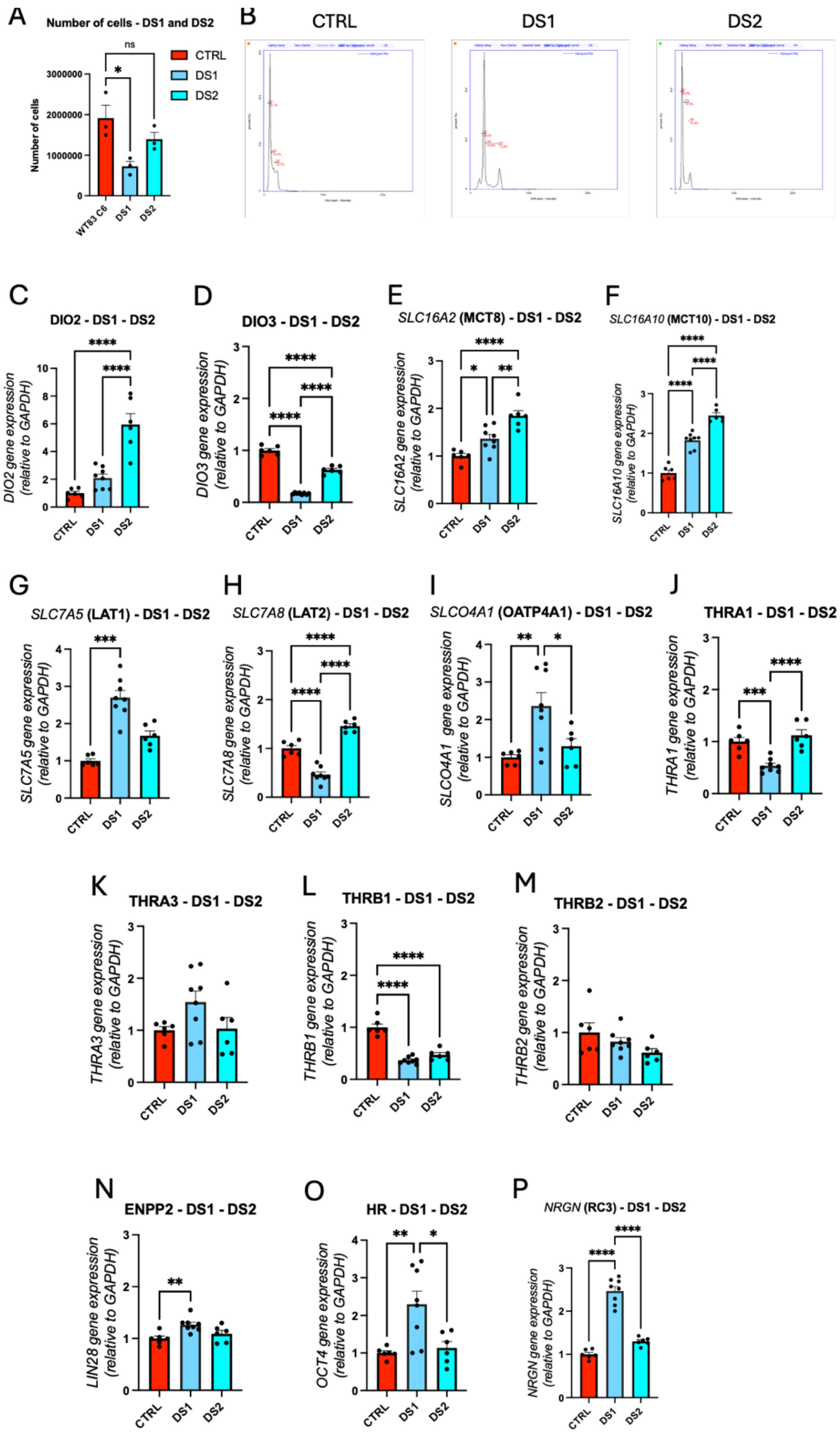

Figure S3. Extended analysis of cell cycle, THs pathway, and AD-related gene expression in DS hid-NPCs: (**a**) Live cell counts for control and DS1 and DS2 hid-NPCs. N=3 experiments (replicates); (**b**) Cell cycle graph; (**c-d**) Relative gene expression (RT-qPCR) of deiodinase *DIO2*, and *DIO3*. N=6 (control), 6 (DS1), and 6 (DS2) replicates; (**e-i**) Relative gene expression (RT-qPCR) of THs transporters

SLC16A2, *SLC16A10*, *SLCO4A1*, *SLC7A5*, and *SLC7A8*. N=6 (control), 6 (DS1), and 6 (DS2) replicates; (**j-m**) Relative gene expression (RT-qPCR) of THRs *THRA1*, *THRA3*, *THRB1*, and *THRB2*. N=6 (control), 6 (DS1), and 6 (DS2) replicates. (**n-p**) Relative gene expression (RT-qPCR) of TH-responsive genes NRGN, ENPP2, and HR. N=6 (control), 6 (DS1), and 6 (DS2) replicates. * $p < 0.05$, ** $p < 0.01$, *** $p < 0.001$, **** $p < 0.0001$.

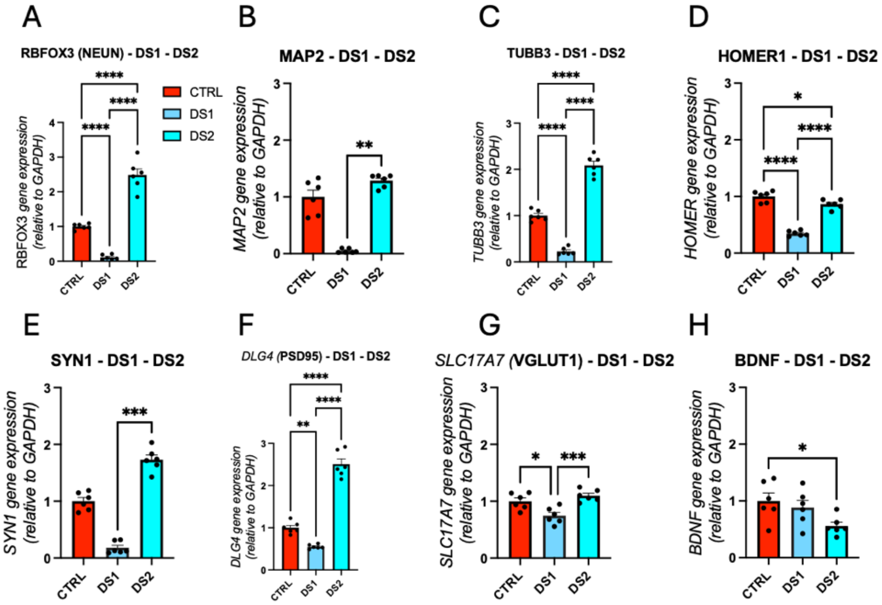

Figure S4. Extended analysis of hid-Neuron markers gene expression: (**a-h**) Relative gene expression (RT-qPCR) of hid-Neuron markers *RBFOX3*, *MAP2*, *TUBB3*, *HOMER1*, *SYN1*, *DLG4*, *SLC17A7*, *BDNF*, and *CTIP2*. N=6 (control), 12 (DS) replicates. N=6 (control), 6 (DS1), and 6 (DS2) replicates. * $p < 0.05$, ** $p < 0.01$, *** $p < 0.001$, **** $p < 0.0001$.

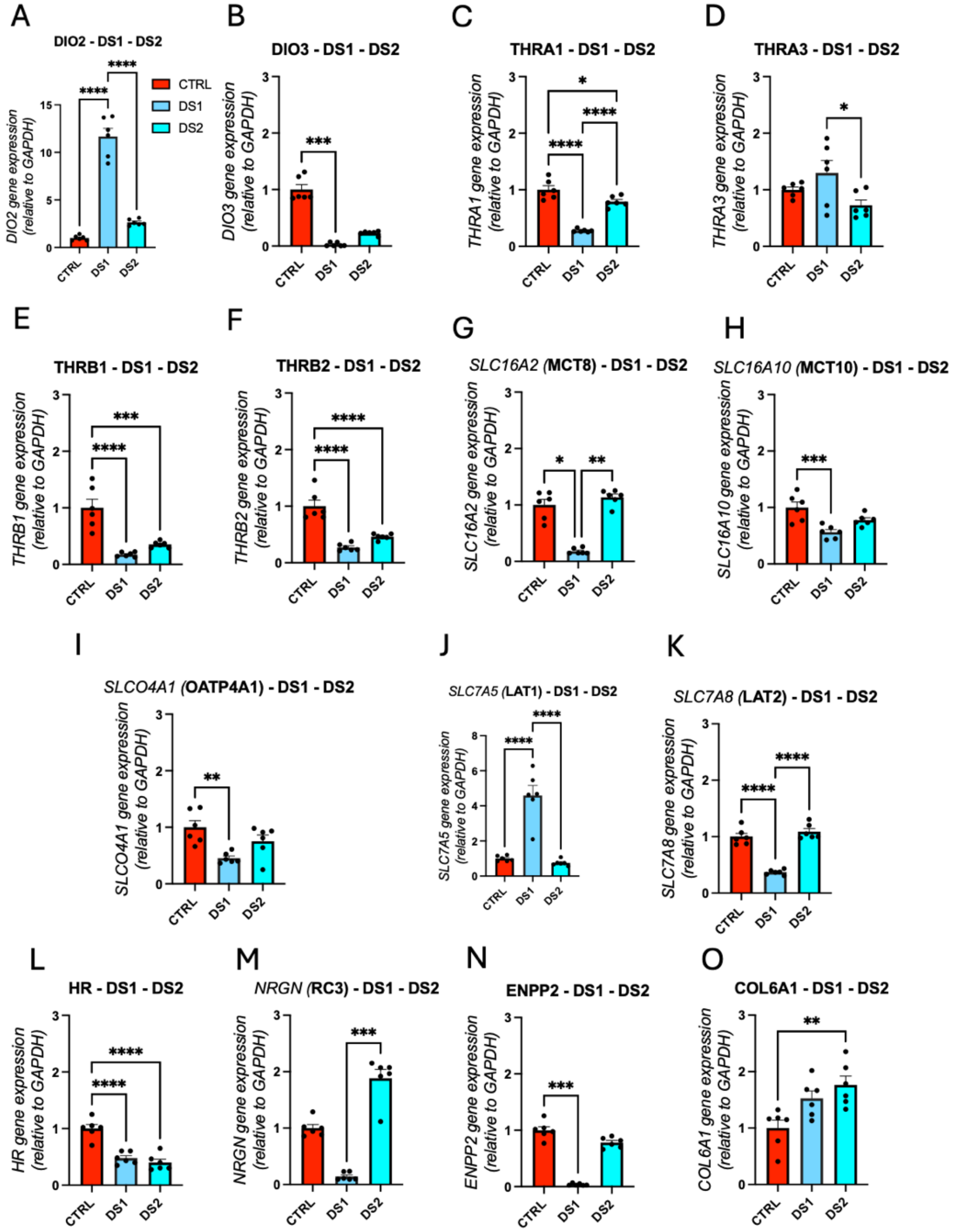

Figure S5. Extended analysis of gene expression of the THs pathway in DS hid-Neurons: (**a-b**) Relative gene expression (RT-qPCR) of deiodinase *DIO2* and *DIO3*. N=6 (control), 6 (DS1), and 6 (DS2) replicates; (**c-f**) Relative gene expression (RT-qPCR) of THRs *THRA1*, *THRA3*, *THRB1*, and *THRB2*. N=6 (control), 6 (DS1), and 6 (DS2) replicates. (**g-k**) Relative gene expression (RT-qPCR) of THs transporters *SLC16A2*, *SLC16A10*, *SLCO4A1*, *SLC7A5*, and *SLC7A8*. N=6 (control), 6 (DS1), and 6 (DS2) replicates; (**l-o**) Relative gene expression (RT-qPCR) of TH-responsive genes *NRGN*, *ENPP2*, *HR*, and *COL6A1*. N=6 (control), 6 (DS1), and 6 (DS2) replicates. * $p < 0.05$, ** $p < 0.01$, *** $p < 0.001$, **** $p < 0.0001$.

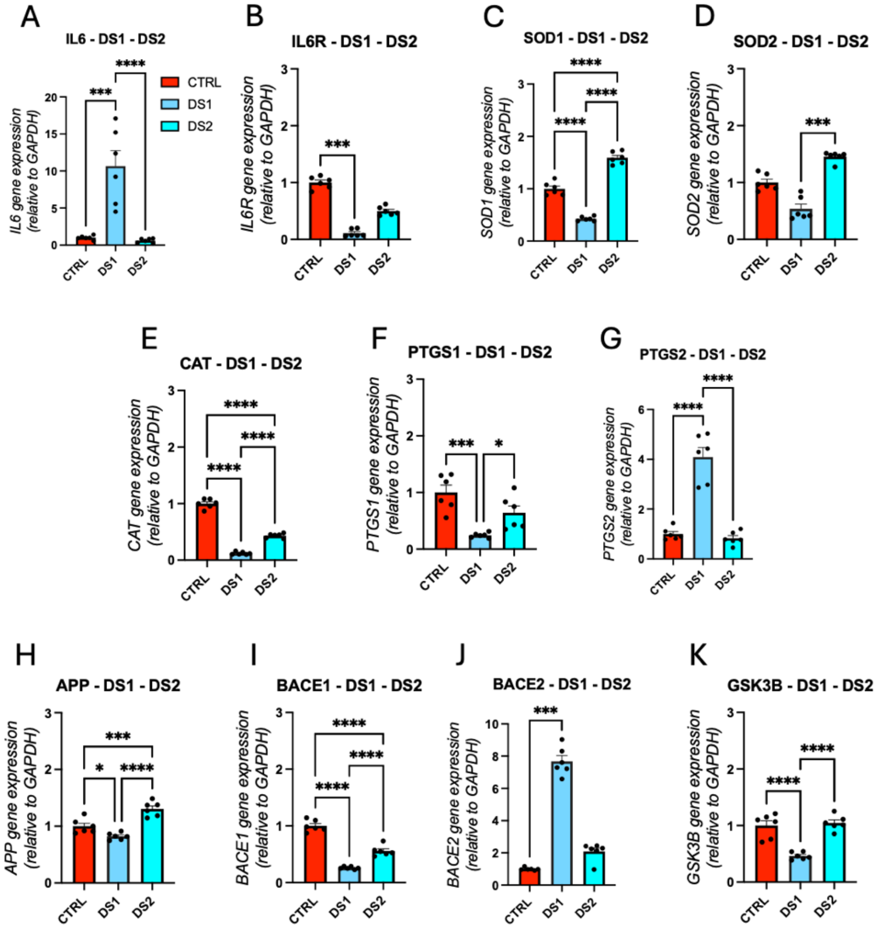

Figure S6. Extended analysis of gene expression of Alzheimer's-related genes: (**a-b**) Relative gene expression (RT-qPCR) of *IL6* and *IL6R*. N=6 (control), 6 (DS1), and 6 (DS2) replicates; (**c-g**) Relative gene expression (RT-qPCR) of oxidative stress genes *SOD1*, *SOD2*, *CAT*, *PTGS1*, and *PTGS2*. N=6 (control), 6 (DS1), and 6 (DS2) replicates; (**h-k**) Relative gene expression (RT-qPCR) of Alzheimer's-related genes *APP*, *BACE1*, *BACE2*, and *GSK3B*. N=6 (control), 6 (DS1), and 6 (DS2) replicates. * $p < 0.05$, *** $p < 0.001$, **** $p < 0.0001$.

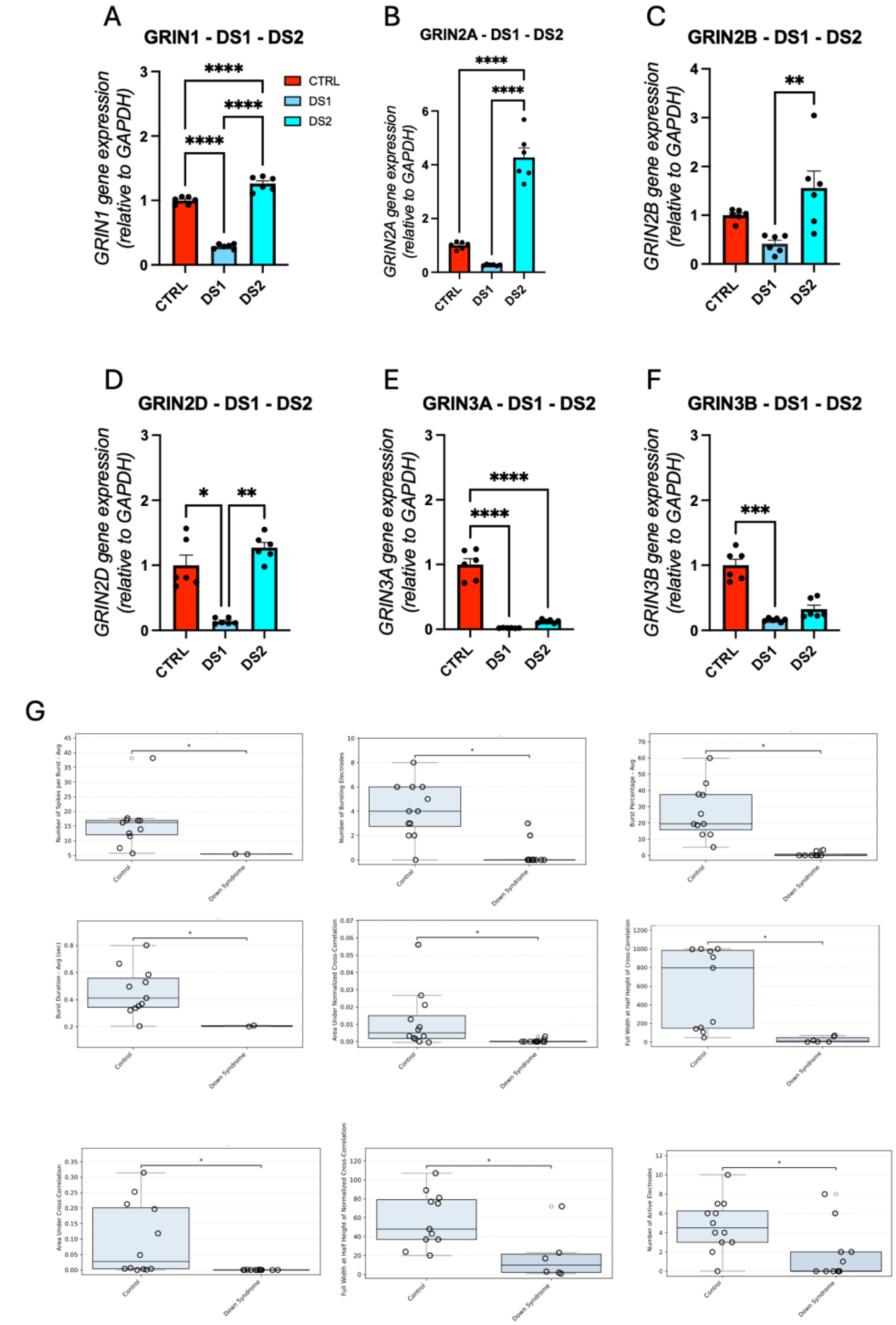

Figure S7. Extended analysis of synaptic deficits in Down syndrome hid-Neurons: (**a-f**) Relative expression (RT-qPCR) of NMDA receptor subunits *GRIN1*, *GRIN2A*, *GRIN2C*, *GRIN2D, GRIN3A,* and *GRIN3B*. N=6 (control),), 6 (DS1), and 6 (DS2) replicates; (**g**) Representative complementary graphics. * $p < 0.05$, ** $p < 0.01$, *** $p < 0.001$, **** $p < 0.0001$.
